# Hybrid analysis of photovoltaic energy data containing excessive structural zeros

**DOI:** 10.1038/s41598-026-58719-0

**Published:** 2026-06-22

**Authors:** Yasin Altinişik, Demet Aydin, Vedat Esen, Taner Dindar, Ali Samet Sarkin

**Affiliations:** 1 Department of Data Science and Analytics, Faculty of Science and Literature, Sinop University, Sinop, Turkey; 2 Department of Research Methods and Statistics, Institute of Psychology, University of Hamburg, Hamburg, Germany; 3 Department of Electric Electronics Engineering, İstanbul Topkapi University, 34087 Istanbul, Turkey; 4 Department of Electronic and Automation, Ankara University, Ankara, Turkey; 5 Department of Electrical and Energy, Osmaniye Korkut Ata University, 80750 Osmaniye, Turkey

**Keywords:** Hybrid modeling, Hurdle models, Structural zeros, Machine learning, Photovoltaic power forecasting, Energy science and technology, Engineering

## Abstract

Photovoltaic (PV) power generation plays a critical role in the global transition toward sustainable energy systems. However, accurate PV power forecasting remains challenging due to the non-stationary nature of PV power time series and the presence of structural zeros. Most existing studies rely on parametric models or treat zero inflation as a secondary issue. In this study, a hybrid hurdle modeling framework is implemented to explicitly account for both structural zeros and continuous positive power values through a two-part structure. The zero component is modeled using Bayesian logistic regression (BLR), random forest classifier (RFC), and support vector classifier (SVC). The positive values are analyzed using the parametric models Gamma regression (GR), Log-normal regression (LNR), and Weibull regression (WR) and the non-parametric machine learning (ML) techniques random forest regression (RFR), extreme gradient boosting (XGBoost), and support vector regression (SVR). The proposed framework is validated using the target variable active power (AP, kW) based on a real-world data from a 110 kWe (129.6 kWp) PV plant located in Çaycuma, Zonguldak, Türkiye. Four metrics were used to compare the predictive performances of the models: mean squared error (MSE), mean absolute (scaled) error (MAE and MASE), and the coefficient of determination ($\:{R}^{2}$). Among the 18 distinct hurdle models, the lowest error values and the highest $\:{R}^{2}$ were obtained when the positive component was modeled by either RFR, such as BLR-RFR (MSE = 67.425 kW, MAE = 3.974 kW, MASE = 0.615, $\:{R}^{2}$ = 0.899) or XGBoost, such as RFC-XGBoost (MSE = 69.252 kW, MAE = 4.075 kW, MASE = 0.630, $\:{R}^{2}$ = 0.898). The findings indicate that hybrid modeling approaches where the zero component is modeled using BLR, RFC, or SVC, and the positive component is modeled with either RFR or XGBoost provide a reliable framework for PV power forecasting, when predicting continuous data characterized by excessive structural zeros.

**Supplementary Information:**

The online version contains supplementary material available at https://doi.org/10.1038/s41598-026-58719-0.

## Introduction

The use of renewable energy sources is on an increasing trend all over the world due to the depletion of fossil fuels and increasing concerns about global warming^1^. Photovoltaic (PV) systems, which convert sunlight directly into electrical energy, are considered the most effective solution in the energy production sector among renewable energy sources because they are clean, cheap and useful^2^. As countries embrace efforts to minimize their carbon footprint, they continue to rapidly add capacity to generate electricity from renewable energy. Solar and wind energy account for 96% of this capacity increase. Solar PV alone accounts for two-thirds of all new renewable electricity technologies. Considering this information, PV is expected to have the largest share in electricity production, which is indispensable for societies in the coming years^3^. Therefore, power plants using PV systems must have stable and reliable power generation. However, electricity production from solar energy varies due to solar radiation, meteorological conditions and seasonal fluctuations. These fluctuations can occur unexpectedly and have a significant impact on how much electricity the PV power plant produces. Additionally, the variability of solar energy production makes it difficult to integrate energy sources and maintain a stable electricity grid. Integration of large-scale PV plants into the electricity grid is an important and difficult issue for electricity operators. Electricity operators have to balance power production and consumption to prevent energy waste and not be penalized due to instability in the electricity grid. They need sophisticated forecasting methods that can precisely predict short-term variations in power output in order to guarantee a steady energy supply in spite of these quick changes^4–6^. Recently, with new technologies, the field of PV power forecasting has developed strongly and many forecasting models have been put forward. The forecasting approaches of these models derive algorithms that take this unpredictability into account to maximize the performance of PV systems^7^.

The motivations driving this research are the desire to solve the problems encountered in PV power forecasting, such as weather dependency, spatial variability, data limitations, and scaling difficulties. Overcoming these challenges requires advanced models, high-quality data, and robust computational methods. Forecasting PV power with high accuracy is crucial for grid stability, proper energy management, and grid integration with renewable energy. In addition, the success of the forecasts allows balancing electricity supply and demand and minimizing grid outages caused by solar energy variability. It helps in optimizations in energy storage systems, corrects the planning of different power sources, and improves market operations by providing reliable forecasts for energy trading. In addition, PV forecasting reduces operational costs, supports regulatory compliance, and maximizes the use of renewable resources.

Numerous studies have been conducted on PV power forecasting techniques, with physics-based approaches based on PV power models widely recognized as one of the leading methods. In addition to these approaches, statistical methods have also attracted considerable attention. Within the statistical forecasting framework, techniques such as time series analysis, machine learning (ML), and deep learning (DL) are commonly used. In recent years, hybrid approaches have gained increasing importance for further improving forecasting accuracy and reliability. These hybrid methods aim to enhance the predictive performance of both physical and statistical models^8–11^.

Some of the statistical approaches estimate future PV power output using time-series data as input information^12–14^. Time series models such as autoregressive moving average (ARMA)^15,16^, autoregressive integrated moving average (ARIMA)^17,18^ and seasonal autoregressive moving average (SARIMA)^19,20^ have proven effective for short term forecasting in large scale PV power plants^21^. Due to their ability to handle nonlinear and heterogeneous inputs, ML models such as artificial neural networks (ANNs)^22,23^ random forest regression (RFR)^24^, support vector regression (SVR)^25^, Gaussian process regression (GPR)^26,27^, and extreme learning machines (ELMs)^28,29^ are widely used in PV power estimation^4,30^. Moreover, methodologies such as RFR, ensemble regression trees, and GPR provide enhanced stability and reliability in predictive performance^31–33^.

Substantial limitations have been detected in some recent hybrid ML studies for PV power forecasting. Asiedu et al.^34^ used data from a grid-connected solar power plant to develop a hybrid prediction system based on RFR, ANNs, and extreme gradient boosting (XGBoost)^35^. The authors developed three hybrid models, namely XGBoost-RFR, ANNs-RFR, and ANNs-XGBoost-RFR, and showed that these models outperform single-stage ML techniques such as linear, Ridge, and Lasso regressions, along with RFR, XGBoost, and ANNs. Nevertheless, the study is subject to several significant limitations. The PV power forecasting was carried out over four time frames, ranging from a single day to one month. Due to this focus on short time horizons, the hybrid models utilized in the analysis remain limited in terms of capturing seasonal variations in PV power forecasting. Additionally, the authors state in the conclusion that the models utilized only three features (irradiation, temperature, and time), thereby significantly restricting the generalizability of their findings on PV power forecasting. Saxena et al.^36^ developed a hybrid model by combining the K-Nearest Neighbors (KNN)^37,38^ model with SVR. The authors demonstrated that KNN-SVR outperforms the traditional long short-term memory (LSTM) technique, achieving an accuracy level of 98%. However, while the research examined the influence of factors including temperature, sunshine duration, solar radiation, and time on solar PV power forecasting, it fails to consider the effects of environmental elements such as wind direction, wind speed, and humidity. Additionally, the study focused exclusively on the performance of the KNN-SVR hybrid model, without considering alternative hybrid configurations. Furthermore, the inherent nature of the employed hybrid model does not have the ability to detect seasonal impacts. In a similar vein, various techniques documented in the literature do not focus on modeling seasonal effects. Esen et al.^39^ employed regression-based models for short-term PV power forecasting owing to their robustness, interpretability, and competitive performance on limited datasets. Ren et al.^40^ reported that a quantile regression approach based on DL effectively captures uncertainty in short-term PV energy predictions, providing reliable probabilistic predictions while maintaining competitive point prediction accuracy. In their study combining GPR with Bayesian hyperparameter adjustment, Islam et al.^26^ obtained accurate and reliable short-term PV power predictions by explicitly modeling uncertainty and maintaining robustness across limited datasets.

The current study demonstrates several notable strengths over the contemporary hybrid ML techniques for PV power forecasting discussed above. When modeling PV power, we account for seasonal variations by incorporating seasonal components into our model. As will be discussed later, in addition to these seasonal components, the model contains various environmental factors influencing PV power generation. Moreover, datasets for PV power forecasting are typically characterized by excessive structural zeros which, unlike sampling zeros, have no potential to take on a different value. However, recent studies often treat zero-inflation as a nuisance factor, despite its potential to exert detrimental effects on parameter estimates. In this regard, particular attention has been paid to the modeling of the structural zeros in the data.

The novelty of the study stems from the fact that it is one of the initial investigations to explicitly integrate structural zeros into the modeling framework. The recommended framework involves hurdle models that serve as specific instances of two-stage models. The application of hurdle models in the domain of PV power forecasting is quite limited. Gillingham and Tsvetanov^41^ examine the demand for solar PV power systems using a hurdle model to analyze the related data containing excess zeros. Kim et al.^42^ enhance the hurdle modeling methodology for urban PV power forecasting by establishing a two-stage ML approach. Neither of the two aforementioned studies above aligns perfectly with the objectives of this study. Unlike the goals of these studies, our research seeks to model seasonal and environmental impacts on PV power forecasting by addressing the structural zeros in the data. Another innovative feature of our research is that the hybrid modeling approach we utilize to address structural zeros remains effective in scenarios where maximum likelihood estimates cannot be obtained due to non-convergence and/or separation problem^43^. The study employs techniques that mitigate these problems when dealing with structural zeros in the data. In summary, the current study aims to simultaneously achieve the following objectives: (i) Capturing seasonal effects in PV power forecasting, (ii) Evaluating the effects of environmental factors (and their interactions) in PV power forecasting, (iii) Accounting for structural zeros inherently present in the data, (IV) Dealing with non-convergence and separation problems when incorporating structural zeros into the analysis procedure. To accomplish these tasks simultaneously, we utilize a comprehensive modeling framework containing 18 different two-stage hurdle models.

Many single-stage parametric models such as Gamma regression (GR)^44^, Log-normal regression (LNR)^44^, and Weibull regression (WR)^45^ are not designed to handle data containing (excessive) structural zeros. This circumstance evidently showcases the need for two-stage hybrid modeling in parametric models, when the model under consideration cannot deal with structural zeros. Additionally, our findings in this study indicate that the two-stage hybrid models we implemented can attain results that are either comparable to or superior to those of ML techniques like RFR^24^, XGBoost^35^, and support vector regression (SVR)^46^. Therefore, our framework enhances the reliability of the analysis, especially in cases where zero values are commonly observed. Although the empirical application focuses on PV power forecasting, the proposed framework can be applied to various renewable energy studies and other fields where structural zeros are common.

Our hybrid modeling approach is composed of two primary components: zero component and positive component. Hybrid modeling exhibits flexibility in that it permits the incorporation of various techniques for modeling structural zeros and positive values in the data independently. The zero component is modeled by Bayesian logistic regression (BLR)^47^, random forest classifier (RFC)^24^, and support vector classifier (SVC)^46^. These approaches are used to model the occurences of structural zeros due to their suitability for binary outcomes, providing computational efficiency and stable estimation in our hybrid modeling framework. The solutions provided by these approaches are important especially when the classical maximum likelihood estimation fails to converge or encounters the separation problem^43^. The positive component is examined through the parametric models GR, LNR, and WR, as well as the non-parametric ML techniques RFR, XGBoost, and SVR. The evaluation process illustrates that integrating BLR, RFC, and SVC with these models or ML techniques yields impressive predictive performance. Conducting a thorough comparative assessment of different forecasting accuracy measures allows for a structured evaluation of the utilized hybrid modeling strategy. In this manner, the content of the study is enhanced through the inclusion of a diverse set of characteristics into the analysis of PV power forecasting data.

The proposed hybrid hurdle modeling framework provides several practical implications for decision-making in photovoltaic energy management. By explicitly modeling structural zeros (e.g., nighttime non-production) separately from positive generation values, the approach enables more accurate production forecasting under zero-inflation conditions. Improved forecasting performance can support short-term operational planning, grid balancing strategies, and energy trading decisions. Furthermore, the enhanced modeling of variability and extreme production values contribute to more reliable risk assessment and capacity planning. Overall, the framework offers a data-driven tool that can assist energy managers and system operators in optimizing resource allocation and reducing operational uncertainty.

## Methodology

**Fig. 1:**
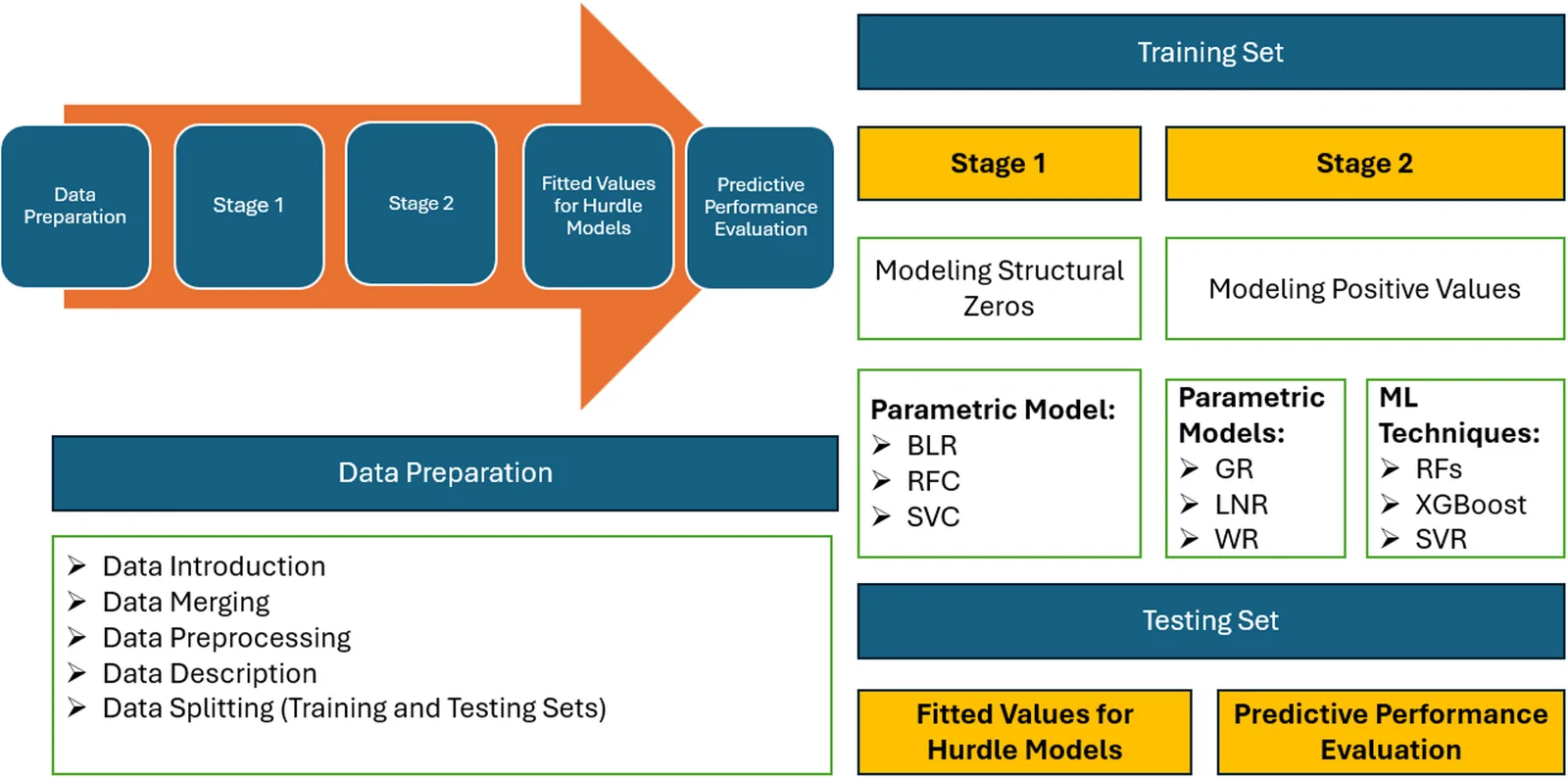
Structure pertaining to the hybrid modeling framework.

This study analyzes continuous data with excess structural zeros using a hybrid modeling framework. Figure 1 depicts the framework structure associated with hybrid modeling. The hybrid modeling strategy is implemented through hurdle models, a specific class of two-stage models. These models separately account for structural zeros and positive values in the PV power forecasting data by employing two distinct sub-models. Following the completion of the data preparation phase, the final data are divided into a training set comprising 80% of the data and a testing set consisting of 20% of the data. In the training set, BLR, RFC, and SVC are used to model structural zeros, while the positive observations are analyzed using both parametric models (i.e., GR, LNR, and WR) and non-parametric ML techniques (i.e., RFR, XGBoost, SVR). In the testing set, the fitted values from the models used in Stages 1 and 2 are combined to evaluate the predictive performance of each hurdle model employed in the research. We commence by introducing the parametric models and non-parametric ML techniques used in Stages 1 and 2. Subsequently, we elaborate on data preparation and the application of hybrid modeling for the training set and testing set of the PV power forecasting data, respectively.

### Modeling structural zeros

At the first stage, we analyze structural zeros in the PV power forecasting utilizing BLR, RFC, and SVC.

#### Bayesian Logistic Regression (BLR)

The classical logistic regression (LR) is widely utilized in modeling binary variables in both statistics and ML disciplines. This technique is applicable when the target variable can solely take on the values of 0 (failure) and 1 (success). It relies on the principle of connecting success probabilities to linear predictors by utilizing the logit link function. The LR model is described as:

1 $${\mathrm{logit}}\left( {\pi _{i} } \right) = X_{i}^{T} {\mathbf{\theta }},$$

where $\:{\pi\:}_{i}$ is the (conditional) probability of success for the ith observation (i = 1, 2, …, n), $\:{X}_{i}$ is the ith row of the design matrix containing the values of the attributes in the model, and **θ** is the vector of model parameters. The logit link function in Eq. (1) relates the success probability for the ith observation with the corresponding linear predictor, that is, $\:{X}_{i}^{T}$**θ**. Here, the logit function is defined as the logarithm of the ratio between the probability of success and the probability of failure (odds), that is, logit($\:{\pi\:}_{i}$) = log ($\:\frac{{\pi\:}_{i}}{1-{\pi\:}_{i}}$).

As will be elaborated later, when modeling the structural zeros within the training set of the PV power forecasting data, the classical LR cannot be utilized in our context due to issues like non-convergence and separation problem^43^. Bayesian logistic regression (BLR)^47^ provides an effective prediction environment in situations where the classical LR encounters such issues. BLR operates on the principle of generating a posterior distribution and utilizing insights gained from this distribution to make predictions. The the posterior distribution in the log scale is defined as follows:

2 $${\text{log P}}(\theta |{\mathrm{y}}){\text{ }} = {\text{ L}}(\theta |{\mathrm{y}}){\text{ }} + {\text{ log P}}(\theta ),$$

where L($\:\theta\:$|y) signifies the information derived from the data, while log P($\:\theta\:$) denotes the prior information. We obtain the estimates of the model parameters in BLR using the *stan_glm* function with *family = binomial(link = “logit”*) option, available in the *rstanarm* package^48^ of *R* statistical software. This function implements a weakly informative N(0, 2.5) prior distribution for regression coefficients as the default option.

#### Random Forest Classifier (RFC)

The RFC^24^ is a widely used non-parametric ensembling technique in the field of ML. In the realm of classification, RFC aims to collate the information obtained from multiple decision trees, and thus, makes more robust predictions compared to a single decision tree. In this regard, RFC represents an advanced version of the decision tree algorithm designed to address more intricate issues. We implement the RFC algorithm using the *randomForest* function of the *randomForest* package^49^ in *R*.

Decision trees are mostly suitable for solving simple problems. The primary reason for this is that intricate data types with many attributes lead the decision tree to branch more frequently (and become increasingly complex) in order to model the target variable, a phenomenon referred to as overfitting^50–52^. RFC is less susceptible to overfitting compared to a single decision tree. In RFC, decision trees (e.g., $\:{n}_{tree}$ = 1000) are generated by drawing independent samples from the training set through non-parametric bootstrapping (with replacement)^53^. In each bootstrap sample, the process of selecting random subsets of features occurs separately for each node within the corresponding decision tree. Based on the data gathered from these bootstrap samples, the classification that receives the highest vote is identified as the final output. Thanks to its non-parametric characteristics and ability to account for non-linear relationships between variables, RFC provides a modeling framework that is remarkably sturdy against issues such as non-convergence and separation, especially when compared to conventional LR models.

#### Support Vector Classifier (SVC)

Support vector machines are applicable for both classification and regression tasks. Within the domain of classification, support vector machines are designated as SVC^44^. It conducts classification in the presence of linear or non-linear relationships between variables utilizing different kernel functions (e.g., linear, polynomial, Gaussian, and sigmoid). In the linear SVC technique, it is assumed that the relationship between the attributes (or features) and the target variable can be elucidated using a linear function, whereas this assumption is relaxed in the non-linear SVC technique. This study employs the Gaussian kernel, which is the most commonly utilized kernel function within SVC, also known as the radial basis function (RBF) kernel^54,55^. We employ SVC with the Gaussian kernel function using the *svm* function of the *e1071* package^56^ in *R* with the *kernel = “radial”* option. The default setting for the kernel parameter was employed, which is the inverse of the total number of attributes (P) in the data. Note that for classification purposes, the target variable must be defined as a factor. Additionally, the “*probability = TRUE*” option must be enabled in *svm*, as it provides the class probabilities necessary to calculate the fitted values in hurdle modeling.

### Modeling positive values using ML techniques

At the secondary stage, we investigate positive values in the PV power forecasting data using both parametric models and non-parametric ML techniques. We employ three non-parametric ML techniques, namely RFR, XGBoost, and SVR.

#### Random Forest Regression (RFR)

Random forests can also be used for both classification and regression purposes. When random forests are employed for regression tasks, they are referred to as RFR^24^. Therefore, similar to RFC, RFR is an ensembling technique based on bootstrap samples for decision trees. One of the key distinctions between RFC and RFR lies in the approach used to extract information from bootstrap samples. In RFR, the predictions regarding the values of the target variable in the testing set are established by averaging the fitted values obtained based on the decision trees. The *randomForest* function of the *randomForest* package^49^ in *R* is also utilized for prediction in RFR. Note that this function automatically executes either classification or regression tasks, depending on the nature of the target variable. Specifically, it conducts classification when the target variable is categorized as a factor, and it performs regression when the variable is numeric.

#### Extreme Gradient Boosting (XGBoost)

The XGBoost^35^ algorithm refers to a boosting technique that successively and dependently trains multiple weak models to develop a strong final model. The study examines decision trees also within the framework of boosting. This approach allows us to compare RFR (using the bagging algorithm) and XGBoost (using the boosting algorithm) on the same basis. The main objective of the bagging technique is to achieve more reliable predictions by minimizing the variance of the outcomes produced by independent decision trees. The boosting approach centers on the sequential construction of weak (biased) decision trees that are interdependent, aiming to achieve a collection of decision trees that is unbiased (or less biased). In this regard, employing RFR and XGBoost within the same framework allows us for the distinct evaluation of prediction performance through both variance and bias reductions. We implement XGBoost using the *xgb.Dmatrix* function in the *xgboost* package^57^ in *R.*

#### Support Vector Regression (SVR)

In the realm of regression modeling, support vector machines are denoted as SVR^44^. SVR is used to reveal linear or non-linear relationships between variables with the assistance of the same kernel functions elaborated for SVC above. We also utilize the Gaussian kernel within the context of SVR, which is the default option. The *svm* function of the *e1071* package^56^ in *R* is also used for regression purposes. To be able to use this function for regression, the target variable must be a numeric vector. As will be elaborated later, we utilize an epsilon-insensitive approach within the SVR framework, where a medium size cost parameter is chosen for the errors. Note that the “*probability = TRUE*” option must be omitted when using the *svm* function for regression purposes.

### Modeling positive values using parametric models

At the secondary stage, we also utilize parametric models to address positive values in the PV power forecasting data. We implement three different parametric models which are GR, LNR, and WR.

#### Gamma Regression (GR)

The GR^44^ model serves as a special form of generalized linear models (GLMs), which is defined as:

3 $${\mathrm{log}}(\mu _{i} ){\text{ }} = Z_{i}^{T} \eta ,$$

where $\:{\mu\:}_{i}$ is the expected value of the positive value for the ith observation (i = 1, 2, ., n), $\:{Z}_{i}$ is the ith row of the design matrix, and $\:\eta\:$ is the vector of regression coefficients^44^. Notably, the design matrices **X** in Eq. 1 and **Z** in Eq. 3 do not necessarily contain the values of the same predictors in the data. The elements of the mean vector are related to the elements of the parameter vector(s) based on the distribution of the target variable. That is, $\:{\mu\:}_{i}$ = $\:{\alpha\:}_{i}{\beta\:}_{i}$ (i = 1, 2, ., n) for GR with $\:{\alpha\:}_{i}$ and $\:{\beta\:}_{i}$ are the ith values of the parameter vectors **α** and **β** containing the values of the shape and scale parameters, respectively. We perform the parameter estimation for GR utilizing the *glm* function in the *stats* package^58^ with *family = Gamma(link = “log”*) option.

#### Log-normal Regression (LNR) and Weibull Regression (WR)

The LNR^44^ and WR^45^ models are the special instances of survival models, which are defined as:

4 $${\mathrm{log}}(T_{i} ){\text{ }} = Z_{i}^{T} \eta + \phi \varepsilon _{i} ,$$

where $\:{T}_{i}$ is the ith value of the target variable (survival time) and ϕ is the scale parameter^59,60^. Here, $\:{\epsilon\:}_{i}$ represents the ith value of the random error which follows a standard normal distribution for the LNR model and a standard extreme value (Gumbel Type I) distribution for the WR model. Similar to GLMs, the components of the mean vector are associated with the components of parameter vector(s) in accordance with the distribution of the target variable. For the LNR model, $\:{\mu\:}_{i}$ = exp($\:{\gamma\:}_{i}$ + $\:\frac{{\sigma\:}_{i}^{2}}{2}$) with $\:{\gamma\:}_{i}$ = $\:{Z}_{i}^{T}\eta\:$ and $\:{\sigma\:}_{i}$ are the ith values of the parameter vectors containing the mean and standard deviations for the logarithm of the survival times. For the WR model, $\:{\mu\:}_{i}$ = $\:{\xi\:}_{i}\:\varGamma\:$(1 + $\:\frac{1}{\nu\:}$) with $\:\nu\:$ (constant) and $\:{\xi\:}_{i}$ are the shape and scale parameters for the ith observation, respectively.

Table 1 displays the ith value of the parameter vector ($\:{\lambda\:}_{i}$), the probability density function (pdf), and the mean vector ($\:{\mu\:}_{i}$) for each of the models used in the second stage of the hurdle models. For each of these distributions, the dependent variable is defined only for positive values. The parameter estimation for the LNR and WR models was carried out through the *survreg* function in the *survival* package^61,62^ in *R*, utilizing the *dist = “lognormal”* and *dist = “weibull”* options, respectively.

**Table 1:** The ith value of the parameter vector ($\:{\lambda\:}_{i}$), probability density function (pdf), and the mean vector ($\:{\mu\:}_{i}$) for the models in the second stage of the parametric hurdle models.

Model	$\:{\lambda\:}_{i}$	$\:\mathbf{P}(\mathbf{y}={y}_{i};\:{\lambda\:}_{i}$)	$\:{\mu\:}_{i}$
Gamma	$\:{\alpha\:}_{i}$, $\:{\beta\:}_{i}$	$\:\frac{1}{\varGamma\:\left({\alpha\:}_{i}\right){\beta\:}_{i}^{{\alpha\:}_{i}}}\:{y}_{i}^{{\alpha\:}_{i}-1}{e}^{-\frac{{y}_{i}}{{\beta\:}_{i}}}$	$\:{\alpha\:}_{i}$ $\:{\beta\:}_{i}$
Log-normal	$\:{\gamma\:}_{i}$, $\:{\sigma\:}_{i}$	$\:\frac{1}{{y}_{i}\sigma\:\sqrt{2\pi\:}}exp(-\:\frac{{(log\:\left({y}_{i}\right)\:-{\gamma\:}_{i})}^{2}}{2{\sigma\:}^{2}})$	exp($\:{\gamma\:}_{i}+\frac{{\sigma\:}_{i}^{2}}{2}$)
Weibull	$\:\nu\:$ (constant), $\:{\xi\:}_{i}$	$\:\frac{\nu\:}{{\xi\:}_{i}}\:{\left(\frac{{y}_{i}}{{\xi\:}_{i}}\right)}^{\nu\:-1}exp(-{\left(\frac{{y}_{i}}{{\xi\:}_{i}}\right)}^{\nu\:-1})$	$\:{\xi\:}_{i}\:\varGamma\:$(1 + $\:\frac{1}{\nu\:}$)

The parametric hurdle models rely on the distribution described below:

5 $$\:\mathrm{P}(\mathrm{y}\:=\:{y}_{i};\:{\uppi\:},\:{\uplambda\:})=\left\{\begin{array}{c}{\pi\:}_{i},\:\:{y}_{i}=0,\\\:(1-{\pi\:}_{i})P\left(\mathrm{y}\:=\:{y}_{i};{\uplambda\:}\right),\:\:{y}_{i}>0,\end{array}\right.$$

where $\:{\pi\:}_{i}$ is the conditional probability of success, stated otherwise, the probability of obtaining a structural zero in the first stage in Eq. (1), $\:\lambda\:$ is the parameter vector in the second stage, and $\:\left(1-{\pi\:}_{i}\right)$ × $\:P\left(y=\:{y}_{i};\lambda\:\right)$ is the probability of obtaining the continuous value ($\:{y}_{i}$) in the second stage^63–65^. Table 2 displays the parametric hurdle models employed in the study, specifically, the hurdle Gamma regression (HGR)^63,64^ hurdle Log-normal regression (HLNR)^65,66^, and hurdle Weibull regression (HWR)^64,65^ models, which are derived from the distribution in Eq. (5).

**Table 2:** The parametric hurdle models based on the distribution in Eq. (5).

Model	$\:{\lambda\:}_{i}$	Stage 1 $\:{y}_{i}$ = 0	Stage 2 $\:{y}_{i}$ > 0
Hurdle Gamma^64,65^	$\:{\alpha\:}_{i}$, $\:{\beta\:}_{i}$	$\:{\pi\:}_{i}$	$\:\left(1-{\pi\:}_{i}\right)\:\frac{1}{\varGamma\:\left({\alpha\:}_{i}\right){\beta\:}_{i}^{{\alpha\:}_{i}}}\:{y}_{i}^{{\alpha\:}_{i}-1}{e}^{-\frac{{y}_{i}}{{\beta\:}_{i}}}$
Hurdle Log-normal^65,66^	$\:{\gamma\:}_{i}$, $\:{\sigma\:}_{i}$	$\:{\pi\:}_{i}$	$\:\left(1-{\pi\:}_{i}\right)\frac{1}{{y}_{i}\sigma\:\sqrt{2\pi\:}}exp(-\:\frac{{(log\:\left({y}_{i}\right)\:-{\gamma\:}_{i})}^{2}}{2{\sigma\:}^{2}})$
Hurdle Weibull^65,66^	$\:\nu\:$ (constant), $\:{\xi\:}_{i}$	$\:{\pi\:}_{i}$	$\:\left(1-{\pi\:}_{i}\right)\frac{{\nu\:}_{i}}{{\xi\:}_{i}}\:{\left(\frac{{y}_{i}}{{\xi\:}_{i}}\right)}^{{\nu\:}_{i}-1}exp(-{\left(\frac{{y}_{i}}{{\xi\:}_{i}}\right)}^{{\nu\:}_{i}-1})$

### Data preparation

As depicted in Fig. 1, our data preparation process comprises five different subprocesses: Data introduction, data merging, data preprocessing, data description, and data splitting. In the following subsections, we provide a brief elaboration on each of these subprocesses, respectively.

#### Data introduction

This study conducted a hybrid modeling that involves two different datasets: the UNALLAR Solar Power Plant (SPP) and a meteorological data obtained from the Open-Meteo website.

The UNALLAR SPP has an installed capacity of 110 kWe (129.6 kWp) and is located in the Çaycuma district of Zonguldak province. Çaycuma is located at the coordinates of 41° 25’ 35.004’’ N and 32° 4’ 32.016 E. The data contain the measurements regarding the target variable, which is the total active power (AP, kW) representing the alternating current active power transferred to the grid at the inverter output. This measure plays an important role in calculating the system performance ratio, energy production efficiency, and inverter conversion losses. Its values are typically 2–5% lower than the total direct current (DC, kW) power.

The meteorological data contain 10 different attributes. That is, temperature-2 m (°C, T), relative humidity-2 m (%, RH), wind speed-10 m (km/h, WS), direct radiation (W/m^2^, DR), direct normal irradiance (W/m², DNI), apparent temperature (°C, AT), terrestrial radiation (W/m², TR), wind direction-100 m (°, WD), dew point-2 m (°C, DP) and cloud-cover (%, CC).

#### Data merging

The UNALLAR plant’s monitoring system recorded the measurements for AP each 15 min from January 4, 2021 to January 5, 2025. The values of the attributes specified above for the meteorological data were recorded on an hourly basis from January 1, 2021 to January 1, 2025. In order to work on both datasets mentioned above simultaneously, the ultimate data we possess contain hourly records spanning from January 4, 2021 to January 1, 2025.

#### Data preprocessing

**Fig. 2:**
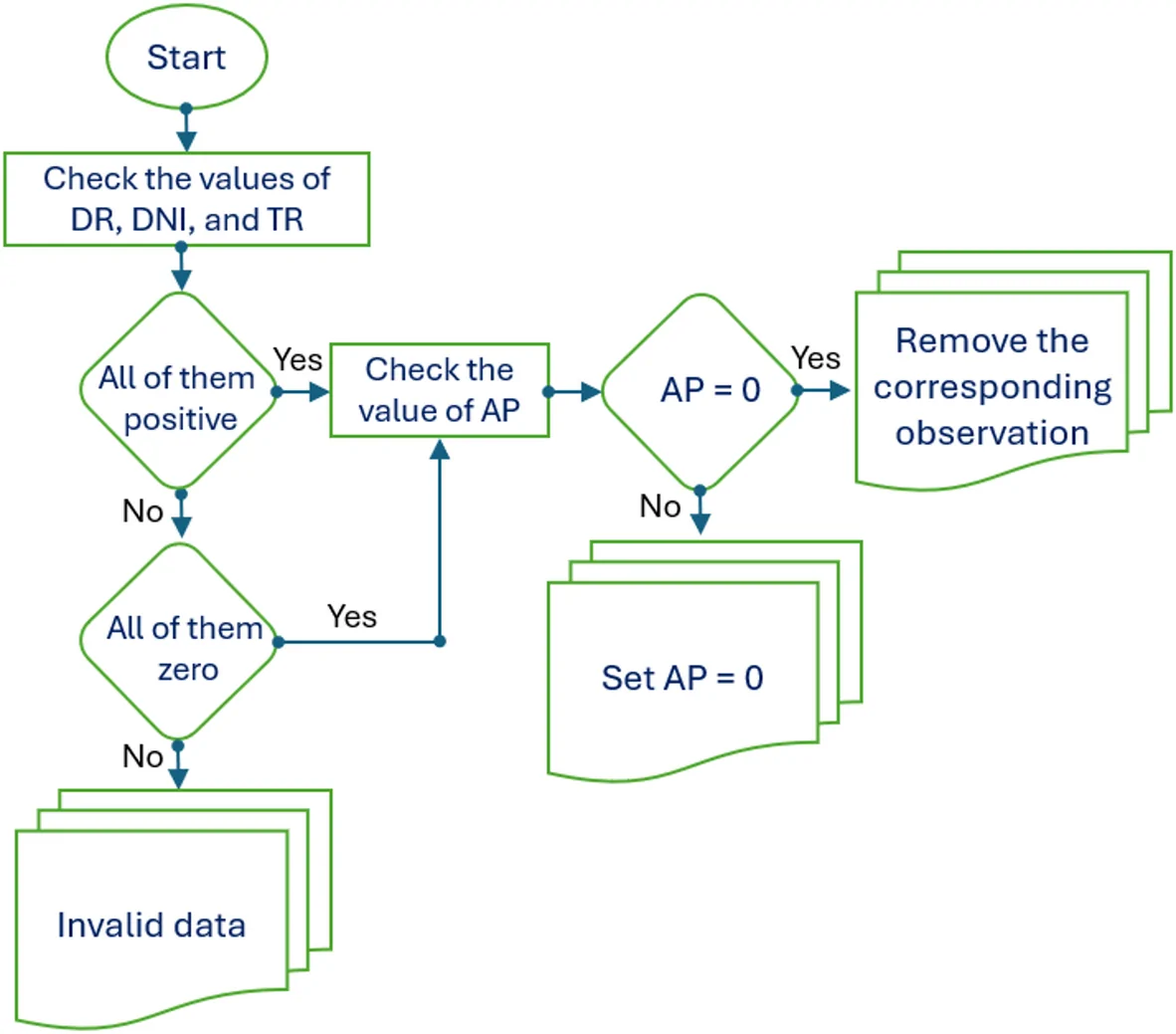
Algorithmic description of the preprocessing rules when dealing with the structural zeros in the PV power forecasting data.

Two preprocessing steps were applied prior to the analysis when dealing with the structural zeros in the data. First, in 86 cases where direct radiation (W/m², DR), direct normal irradiance (W/m^2^, DNI), and terrestrial radiation (W/m^2^, TR) were zero but active power (AP, kW) took small positive values, AP was corrected to zero in accordance with the structural zero mechanism in the data. When DR = 0, DNI = 0, and TR = 0, the corresponding solar AP value is expected to be structurally zero. This is because such a scenario signifies an absence of solar energy, which can occur during nighttime, system outages, equipment failures, or measurement errors. Note that, since TR = 0 denotes absolute zero (-273.15 °C), this condition cannot occur in practice. Second, 829 observations (2.4% of the data) in which DR > 0, DNI > 0, and TR > 0, while AP = 0 were removed, as they were inconsistent with the structural zero mechanism in the data. Because the lack of PV power generation (AP = 0) during daylight hours (DR > 0 and DNI > 0) indicates that there may have been system outages, equipment failures, or measurement errors. It should be noted that these occurences do not signify structural zeros. Conversely, these instances indicate situations in which AP = 0 is observed despite the expectation of AP > 0. After these adjustments, the final data set consisted of 32,943 hourly observations. It is important to note that a decrease in the accuracy of the classification methods (BLR, RFC, and SVC) is observed when these preprocessing steps are omitted. The implementation of these preprocessing steps is also crucial for aligning the data with the concept of structural zeros. This also acts as tangible evidence demonstrating the importance of DR, DNI, and TR in predicting solar PV power. Figure 2 displays the algorithmic description of the preprocessing rules when dealing with the structural zeros in the PV power forecasting data.

It should be emphasized that there is no standardization involved in the two-stage hurdle modeling procedure. Note that the GR, LNR, and WR parametric models necessitate the target variable AP to be positive. In this context, the traditional standardization approach is not suitable for these models, as it produces negative values. Additionally, since RFR and XGBoost are based on decision trees, they generally do not require standardization. Although a standardization procedure for the geometry-based SVR technique (and RFR and XGBoost) was contemplated, it has been observed that this approach significantly distorts the analysis results based on the four evaluation metrics employed in this study. All results regarding the analysis of the unstandardized data can be duplicated by utilizing *set.seed(111)* in *R* statistical software.

### Data description

Descriptive statistics of the processed data are presented in Table 3. Skewness coefficients demonstrate that all variables exhibit departures from zero, indicating that none of the distributions can be characterized as symmetric. The attributes T (S = 0.07), WS (S = 0.99), DR (S = 1.72), DNI (S = 1.04), AT (S = 0.09), TR (S = 0.84), and the target variable AP (S = 1.54) display positively (right) skewed distributions, typically associated with elongated or heavy right tails. Conversely, RH (S = − 0.59), WD (S = − 0.53), DP (S = − 0.23), and CC (S = − 0.05) exhibit negatively (left) skewed distributions, in which the tail extends toward smaller values and most of the observations are concentrated at higher values. The attributes WS (K = 4.74), DR (K = 4.93), and AP (K = 4.22) have kurtosis values above three, reflecting leptokurtic distributions with sharper peaks and heavier tails, suggesting a higher propensity for extreme observations. All the other variables in the data exhibit kurtosis values below three, indicating platykurtic distributions characterized by flatter peaks and lighter tails, consistent with more stable data and fewer extreme values.

**Table 3:** Descriptives of the processed data.

	Mean	Standard deviation	Median	Skewness (S)	Kurtosis (K)	SE	Coefficient of variation (CV)
T	14.98	7.85	14.80	0.07	2.39	0.04	0.52
RH	77.12	16.40	80.00	-0.59	2.36	0.09	0.21
WS	7.43	4.13	6.60	0.99	4.74	0.02	0.56
DR	119.40	199.89	0.00	1.72	4.93	1.10	1.67
DNI	205.98	288.52	0.00	1.04	2.49	1.59	1.40
AT	14.56	9.80	14.00	0.09	2.26	0.05	0.67
TR	332.46	408.81	57.20	0.84	2.24	2.25	1.23
WD	209.76	102.26	209.00	-0.53	2.53	0.56	0.49
DP	10.56	6.66	10.60	-0.23	2.35	0.04	0.63
CC	52.20	42.64	53.00	-0.05	1.24	0.23	0.82
AP	16.33	25.35	0.26	1.54	4.22	0.14	1.55

The coefficient of variation (CV) is a standardized measure used to compare the variability of distributions expressed in different units. Values of CV < 1 indicate low variability, whereas CV > 1 suggests high variability. Moreover, higher CV values further imply that the observations are more widely dispersed around the mean. The greatest values of CV were observed in DR (CV = 1.67), AP (CV = 1.55), DNI (CV = 1.40), and CC (CV = 0.82), respectively, while RH (CV = 0.21) displayed the lowest degree of variability.

**Fig. 3:**
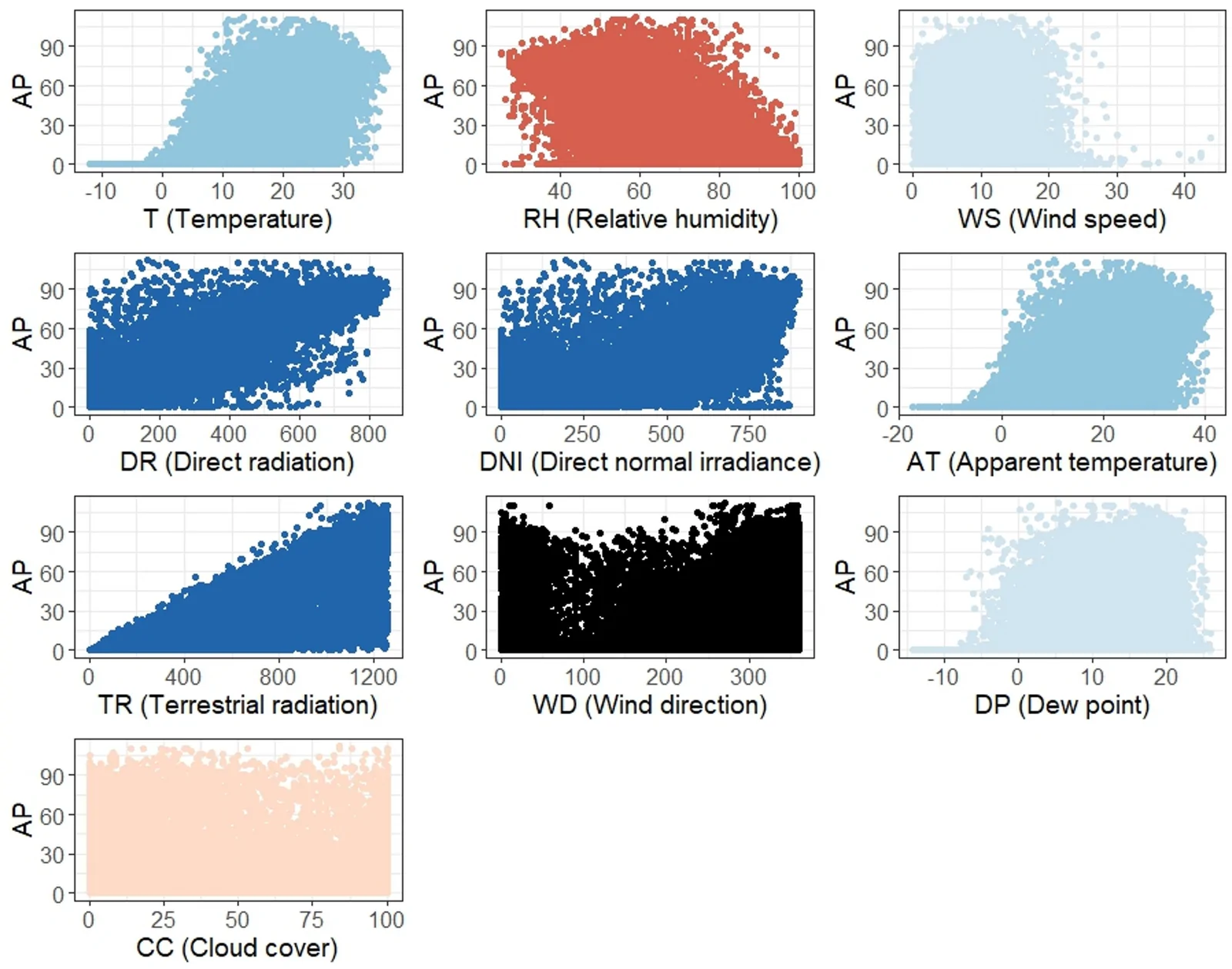
The pairwise relationships between the independent variables and the dependent variable active power (AP) in the processed data.

**Fig. 4:**
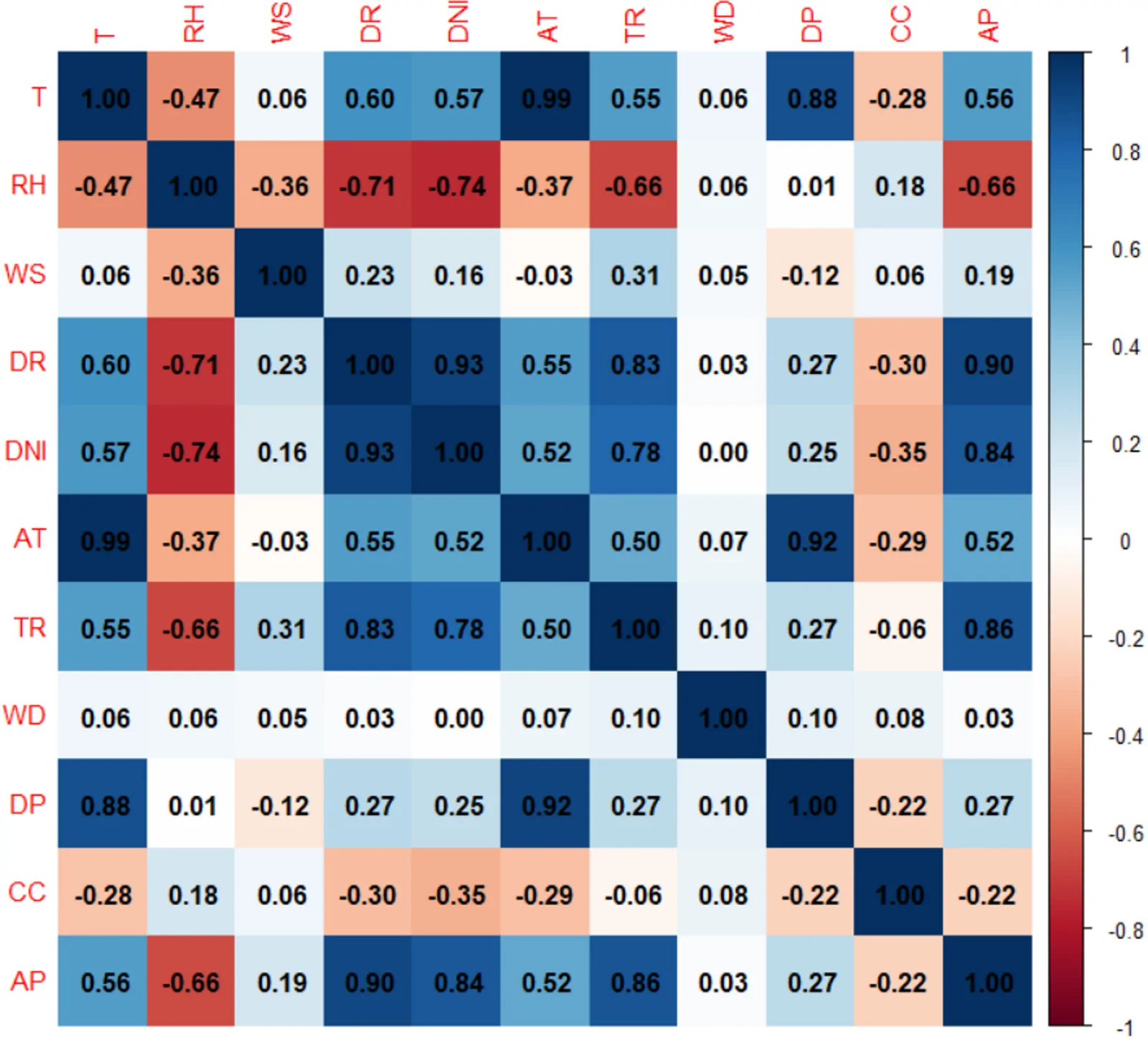
The correlation plot between the variables in the processed data.

Figure 3 displays the pairwise relationships between the attributes and target variable. This figure indicates that only RH exhibits a negative correlation with AP, while all the other variables in the data demonstrate positive correlations with AP. Figure 4 depicts the Pearson correlation coefficients between the attributes and the target variable AP. This figure shows that, in line with the plot in Fig. 3, AP is positively correlated with DR (*r* = 0.90, *p* < 0.001), DNI (*r* = 0.84, *p* < 0.001), and TR (*r* = 0.86, *p* < 0.001) and negatively correlated with RH (*r* = -0.66, *p* < 0.001). There is a weak but statistically significant positive correlation between AP and WD (*r* = 0.03, *p* < 0.001). Note that since there is an almost perfect correlation between T and AT (*r* = 0.99, *p* < 0.001), we utilize T (but not AT) to avoid a potential overfitting problem in our hurdle modeling.

#### Data splitting

To prevent look-ahead bias or data leakage issues, we do not utilize random splitting when forming the training and testing sets. Instead, we utilize the initial 80% of the data for the training set, reserving the remaining 20% for the testing set. The training sample contains 26,354 observations, while the testing set consists of 6,589 observations. The training set observations are utilized to develop the hurdle models addressing both the modeling of structural zeros and the positive values present in the processed data. The observations in the testing set are employed to obtain the combined fitted values for the hurdle models, which are subsequently used to evaluate the predictive performance of these models.

### Application of hybrid modeling in the training set

We utilize the model in Eq. 1 to train the zero component of the hurdle model. The training performance of the zero component is evaluated based on its ability to identify the structural zeros present in the training set in terms of prediction probabilities and confusion matrix. We employ each of the non-parametric ML techniques (i.e., RFR, XGBoost, and SVR) and the parametric models (i.e., GR, LNR, and WR) at the secondary phase of the hurdle modeling in the training set. The performance of the component for positive values is examined by analyzing the relationship between the fitted values and the target variable AP.

#### Stage 1: modeling structural zeros in the training set

The initial phase of hurdle modeling involves incorporating structural zeros into the modeling process. The appropriate integrations of BLR, RFC, and SVC into the analytical process is essential for examining the training set, given that 49.43% of the values for the target variable AP are structural zeros (i.e., 13,027 structural zeros out of a total of 26,354 cases). The LR model in Eq. (1) fails to converge when analyzing the training set. The model also suffers from the separation problem^43^ which causes detrimental effects on the standard errors of the regression coefficients for DR (SE = 45.83) and DNI (SE = 43.01). We address the issues of non-convergence and separation simultaneously by utilizing BLR, RFC, and SVC, respectively.

The BLR model provides the (conditional) probability of success for each observation in the data depending on the positions of the structural zeros (i.e., 0: not a structural zero and 1: structural zero). Consequently, the model is expected to produce high success probabilities for the cases of structural zeros, specifically for ones. In the *rstanarm* package^48^, the BLR model adopts a chain-based strategy (by default 4 chains) involving to produce posterior samples through the Bayesian Markov Chain Monte Carlo (MCMC) algorithm. Table 4 displays the training times for the warm-up and sample procedures associated with each chain.

**Table 4:** Training times for the warm-up and sample procedures associated with each of the four chains in BLR.

	Warm-up	Sample
Chain 1	301.500 s	336.884 s
Chain 2	316.605 s	306.546 s
Chain 3	286.203 s	324.676 s
Chain 4	312.686 s	293.138 s

We implement the Bayesian MCMC using N(0, 2.5) prior distribution for regression coefficents which is the default option in *rstanarm* package^48^. Altinisik^66^ performs a comparative analysis that involves the application of various prior distributions to address the separation problem.

The BLR used to model the structural zeros in the training set is defined as:

6 $$\begin{aligned} {\mathrm{logit}}(\pi _{i} ){\text{ }} & = \theta _{0} + \theta _{1} T_{i} + \theta _{2} RH_{i} + \theta _{3} WS_{i} + \theta _{4} DR_{i} + \theta _{5} DNI_{i} + \theta _{6} TR_{i} + \theta _{7} WD_{i} \\ & \;\;\; + \theta _{8} DP_{i} + \theta _{9} CC_{i} + \theta _{{10}} t_{i} + \theta _{{11}} ~sin\left( {\frac{{2\pi t_{i} }}{P}} \right) + \theta _{{12~}} cos\left( {\frac{{2\pi t_{i} }}{P}} \right), \\ \end{aligned}$$

where $\:{\theta\:}_{0}$ is the intercept term, $\:{\theta\:}_{1}$, $\:{\theta\:}_{2}$, $\:{\theta\:}_{3}$, $\:{\theta\:}_{4}$, $\:{\theta\:}_{5}$, $\:{\theta\:}_{6}$, $\:{\theta\:}_{7}$, $\:{\theta\:}_{8}$, and $\:{\theta\:}_{9}$ are the regression coefficients of variables T, RH, WS, DR, DNI, TR, WD, DP, and CC, respectively, for i = 1, 2, …, n. Here, $\:{t}_{i}$ represents the linear time-trend component (i.e., $\:{t}_{i}$ = i for i = 1, 2, …, n) and $\:{\theta\:}_{10}$ is the corresponding regression coefficient that indicates how the target variable AP varies over time, assuming that the other variables in the model are constant. Similarly, $\:sin\left(\frac{2\pi\:{t}_{i}}{P}\right)$ and $\:cos\left(\frac{2\pi\:{t}_{i}}{P}\right)$ are the seasonal components of the model, where *P* = 4 is utilized to address seasonality, given that the data were collected on an annual basis. Therefore, $\:{\theta\:}_{11}$ and $\:{\theta\:}_{12\:}$ are used to capture periodic fluctuations in AP over time.

**Fig. 5:**
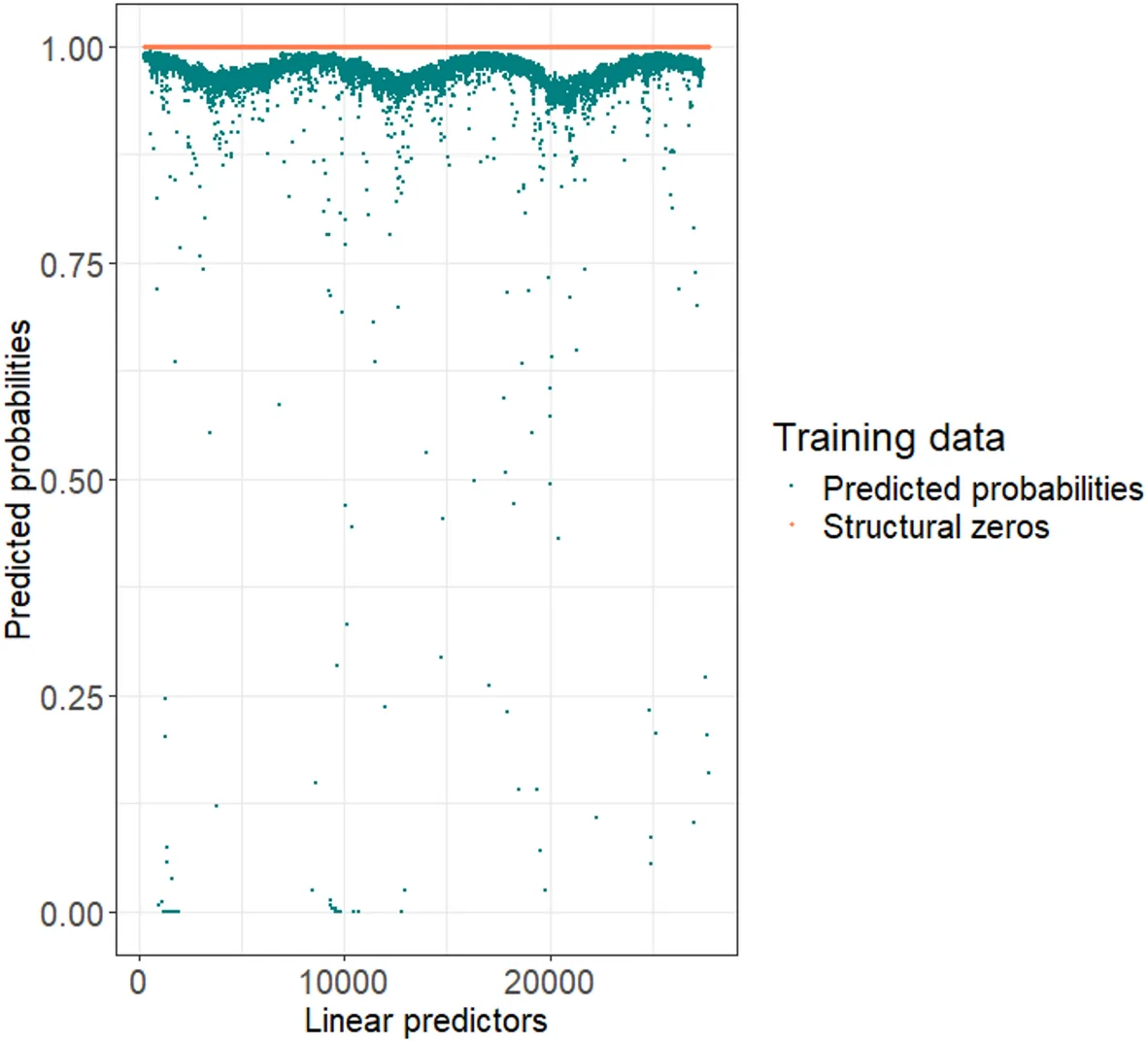
The relationship between the structural zeros in the data and predicted probabilities of the BLR model.

The relationship between the observed structural zeros and the fitted values (predicted probabilities) of the BLR model above is depicted in Fig. 5. The orange dots in the figure indicate the occurrences of structural zeros, namely, ones. The green dots represent the predicted probabilities of observing structural zeros, which are obtained by fitting the model in Eq. 6. The figure reveals that the BLR model predominantly provides high predicted values for the structural zeros. This means that the zero component of our hurdle model is proficient in modeling the structural zeros in the training set.

For both RFC and SVC, the target variable AP along with the identical independent variables from Eq. 6 were utilized. Unlike BLR, RFC and SVC do not utilize regression-based methodologies. While RFC performs classification using entropy-based information theory, SVC conducts classification by examining the degree to which multivariate observations deviate from a decision boundary in a geometric space. Therefore, RFC and SVC directly produce class labels without operating on a probability basis. The duration of training for RFC (i.e., 20.40 s) and SVC (i.e., 35.21 s) is significantly less than that of BLR (see Table 4).

Table 5 displays the confusion matrices used to compare the performances of BLR, RFC, and SVC based on their correct classification rates (CCR). Since BLR is a regression-based approach, the relevant confusion matrix is constructed by designating predicted values greater than 0.5 as one, and those that are 0.5 or lower as zero. There is no cut-off value involved in the construction of the confusion matrices for RFC and SVC. Table 5 shows that RFC exhibits the highest level of accuracy according to CCR, specifically, CCR = $\:\frac{\mathrm{13,219}+\mathrm{12,92}8}{\mathrm{13,219}+99+108+\mathrm{12,928}}$ = 0.992. The lower and upper bounds of the 95% confidence interval of the CCR for RFC are obtained as 0.991 and 0.993, respectively. The Kappa coefficient for RFC is calculated as K = 0.984, signifying a strong model for predicting the structural zeros within the training set. Similar to RFC, high levels of accuracy are obtained for BLR and SVC, with CCR = 0.980 for both. For both BLR and SVC, the corresponding 95% confidence intervals are obtained as [0.978, 0.982]. High values of the Kappa coefficient are also observed for BLR and SVC, with K = 0.960 for both.

**Table 5:** The confusion matrices between the actual and predicted values based on BLR, RFC, and SVC for the training set.

	Predicted values	Status	
		0: Not a structural zero	1: Structural zero
BLR	0	12,869	64
	1	458	12,963
RFC	0	13,219	99
	1	108	12,928
SVC	0	12,853	56
	1	474	12,971

In summary, the results indicate that each of the BLR, RFC, and SVC techniques exhibits satisfactory performance in terms of modeling the structural zeros present in the PV power forecasting data. The primary disadvantage of the BLR technique is considered to be its significantly longer execution time compared to RFC and SVC. Additionally, as mentioned, the confusion matrix for BLR was established under the weakly informative N(0, 2.5) prior distribution and utilized the default cut-off value of 0.5. The sensitivity of the results derived from BLR concerning the choice of prior distribution and cut-off value was not examined, as this falls outside the scope of this study. The fact that the RFC technique provides the highest accuracy in terms of CCR also deters us from undertaking such a comprehensive sensitivity analysis. Note that RFC and SVC utilize identical values of tuned hyperparameters as RFR and SVR, which are elaborated in the next subsection.

#### Stage 2: modeling positive values in the training set

**Fig. 6:**
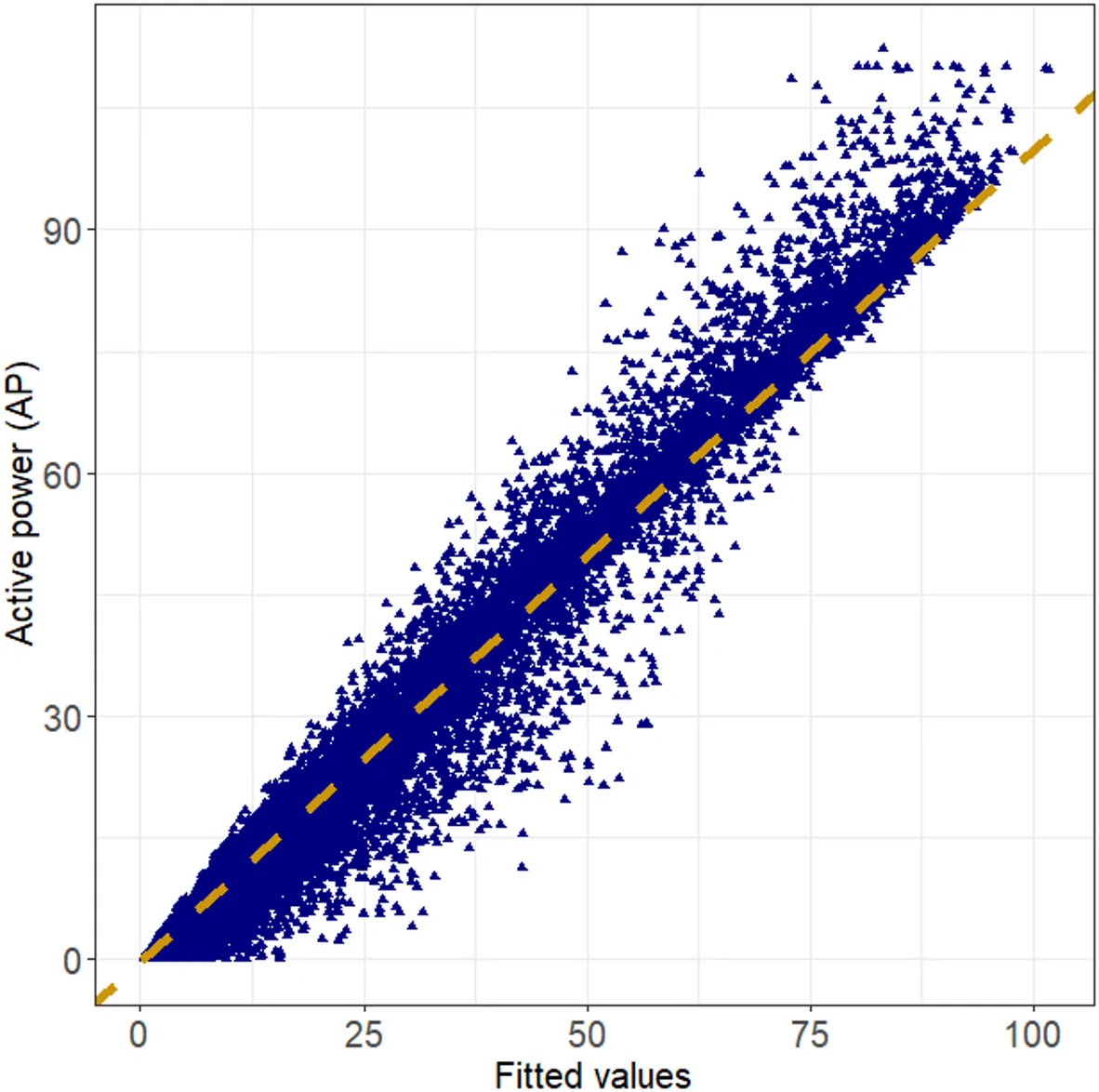
The relationship between the fitted values derived from RFR and the values of AP.

**Fig. 7:**
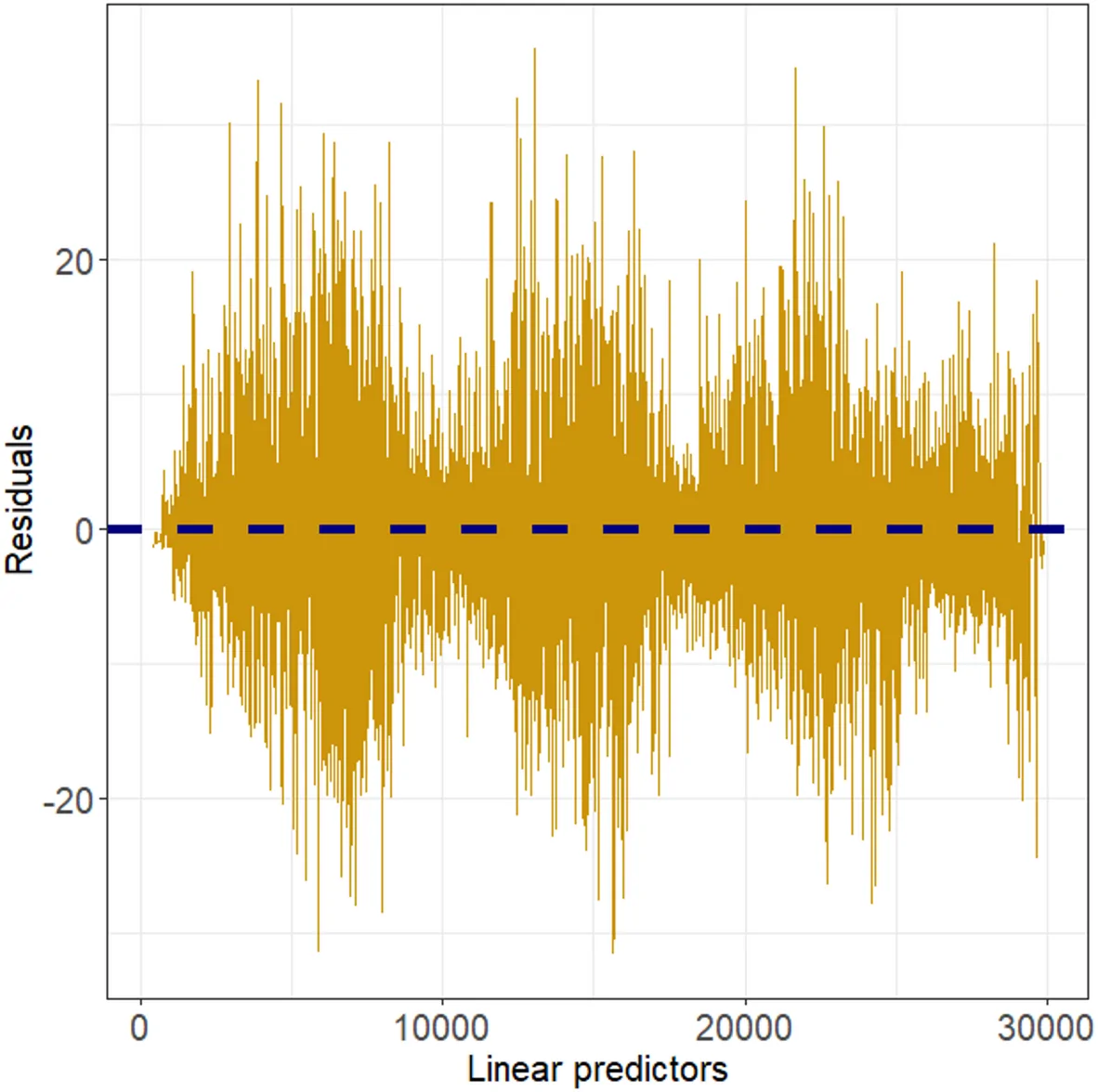
The relationship between the linear predictors and residuals in RFR.

As previously stated, we employ the non-parametric ML techniques RFR, XGBoost, and SVR and the parametric models GR, LNR, and WR for modeling positive values in the data. The same attributes in BLR, RFC, and SVC from the preliminary phase are employed in the execution of RFR, XGBoost, and SVR for modeling positive values. Figure 6 displays the relationship between the fitted values obtained using RFR and the values of the target variable AP. There appears to be a positive linear relationship between the fitted values and AP, suggesting that RFR is effective in predicting the values of AP. Figure 7 shows the relationship between the linear predictors and residuals (the differences between the values of AP and the corresponding fitted values) using RFR. This figure shows that the residuals fluctuate around zero, which implies that there is no systematic error associated with the use of RFR for predicting AP.

**Fig. 8:**
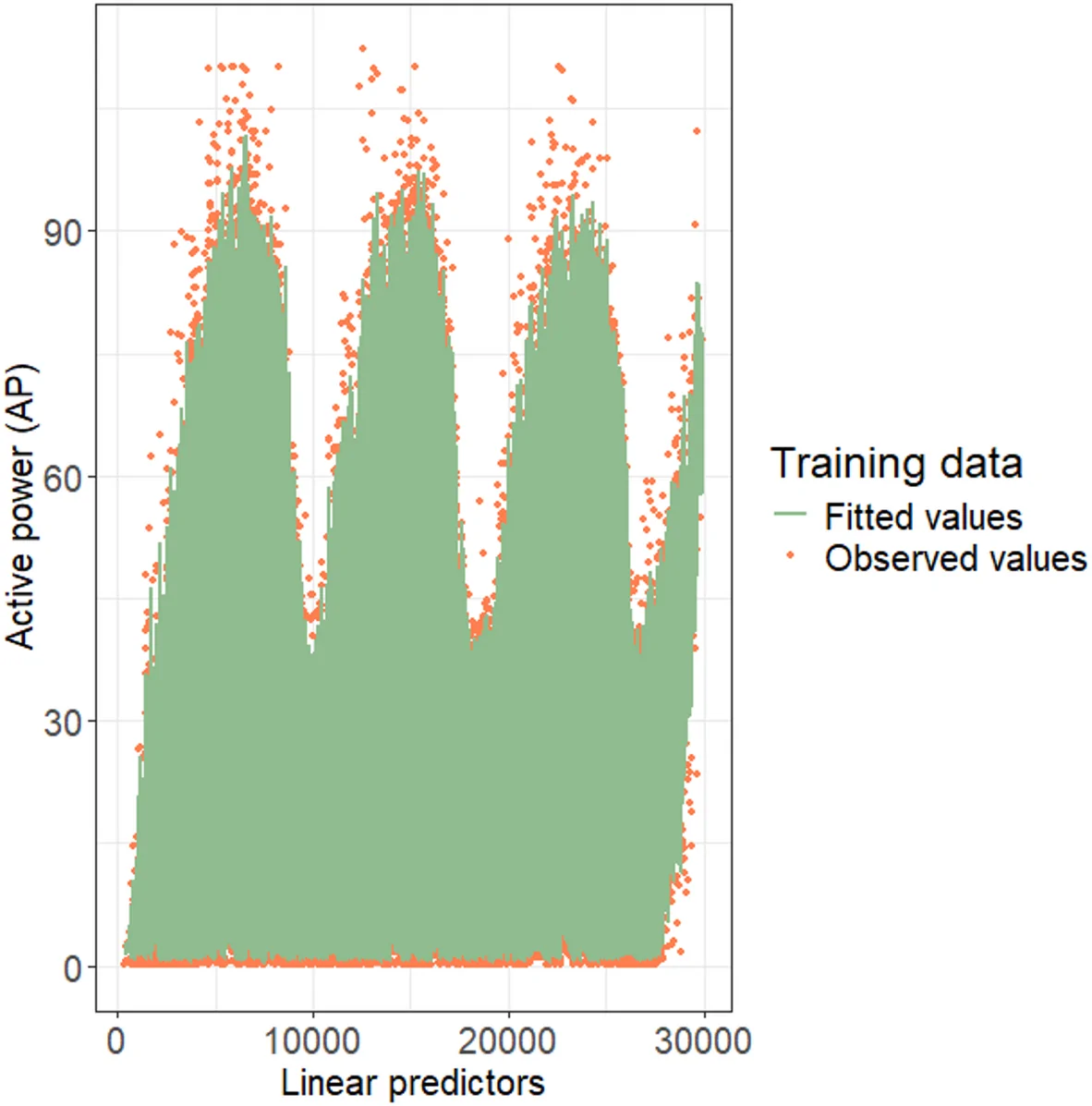
The relationship between the linear predictors and the values of AP using RFR.

Figure 8 depicts the relationship between the linear predictors and the values of AP using RFR. This illustration suggests that RFR is effective in capturing the seasonal impact of the attributes on AP. Figures 6 and 7, and 8 are also obtained by using XGBoost, SVR, GR, LNR, and WR, which are not included here but can be provided upon request.

**Fig. 9:**
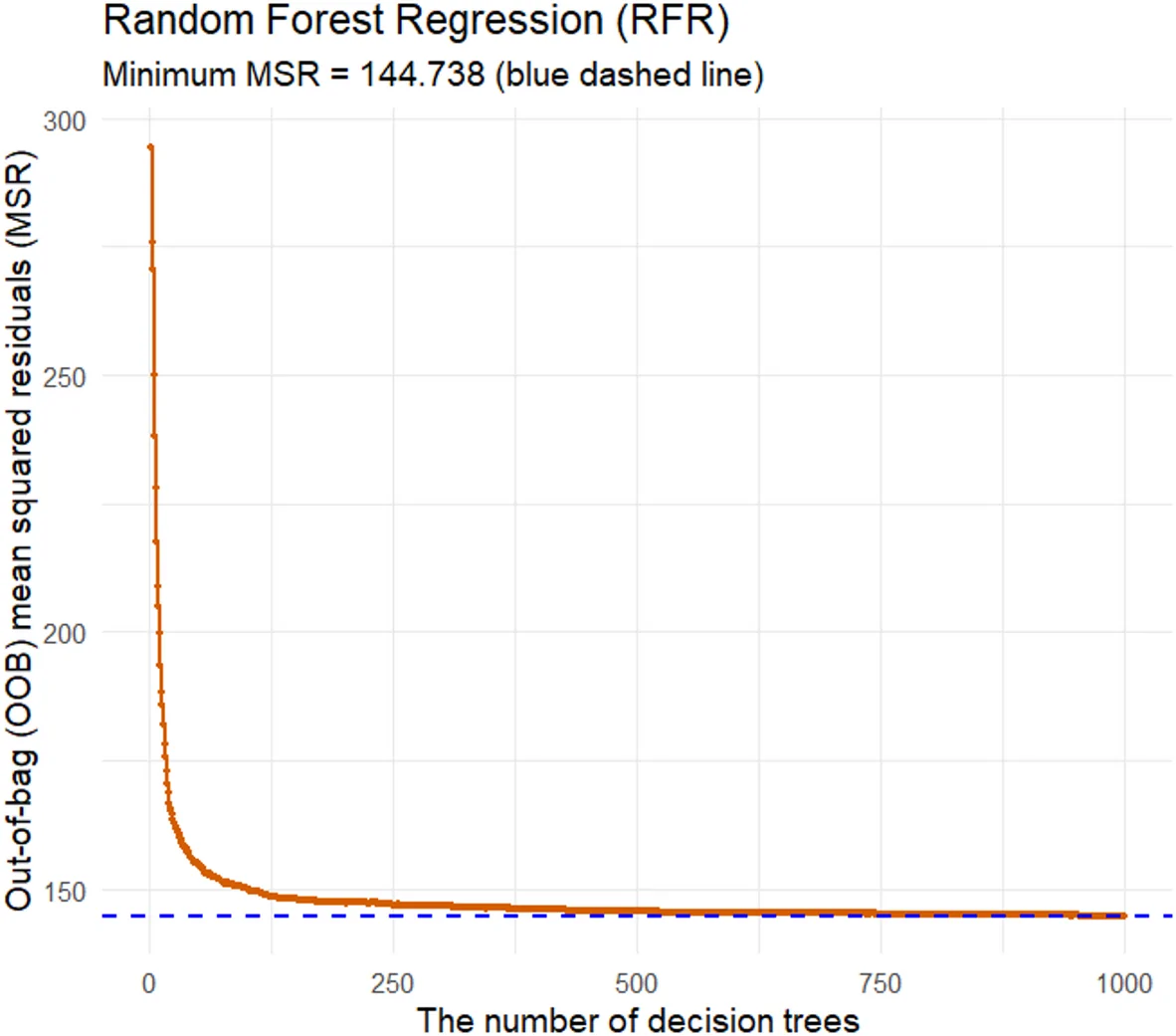
Variation of out-of-bag (OOB) mean squared residuals (MSRs) in relation to the number of trees in RFR.

Table 6 shows the information pertaining to RFR, XGBoost, and SVR, including their corresponding tuned hyperparameters. The table additionally displays the system time necessary to execute the *R* code for each ML technique. It appears that XGBoost and SVR provide analysis results that are considerably quicker than those produced by RFR. In RFR, the mean squared residuals (MSRs) are derived from the out-of-bag (OOB) observations, which refers to the data excluded from the bootstrap sample of the training set for each decision tree (with ntree = 1000). Figure 9 shows how the MSRs vary in relation to the number of decision trees in RFR. This graph illustrates that the MSRs stabilize significantly, particularly after achieving a tree count of 250–300. Thus, the number of trees utilized (i.e., ntree = 1000) is quite sufficient. The minimum value of the MSRs recorded is 144.738 (refer to the blue dashed line in Fig. 9), which serves as an estimate for the MSR in the testing set. In addition, the rapid decrease in the MSRs demonstrates that RFR executes an effective learning in the training set.

**Table 6:** Details regarding RFR, XGBoost, and SVR along with their respective tuned hyperparameters.

Random Forest Regression (RFR)
type: Regression
R package: randomForest
ntree: 1000 (The number of decision trees)
mtry: 3 (The number of attributes to be randomly selected at the split points)
nodesize: 5 (The smallest number of observations that can be contained in a node)
system time: user (130.36 s), system (0.33 s), elapsed (130.90 s)
Extreme Gradient Boosting (XGBoost)
booster: gbtree (Decision tree-based approach)
R package: xgboost
nrounds: 300 (The number of decision trees)
eta: 0.01 (The proportion of errors rectified in the preceding tree in the system)
max_depth: 5 (Maximum depth of decision trees)
subsample: 0.7 (The randomly selected portion of the training set in each decision tree within the system)
colsample_bytree: 0.7 (The randomly selected portion of the attributes in the training set)
system time: user (4.33 s), system (3.67 s), elapsed (1.86 s)
Support Vector Regression (SVR)
type: eps-regression (Epsilon insensitive based approach)
R package: e1071
kernel: radial (Gaussian kernel function is used when determining the values in the kernel matrix)
cost: 1 (A medium size of the cost term is chosen for the errors, which serves as the default option)
epsilon: 0.5 (The chosen maximum magnitude of the errors that are ignored)
system time: user (4.93 s), system (0.01 s), elapsed (4.94 s)

**Fig. 10:**
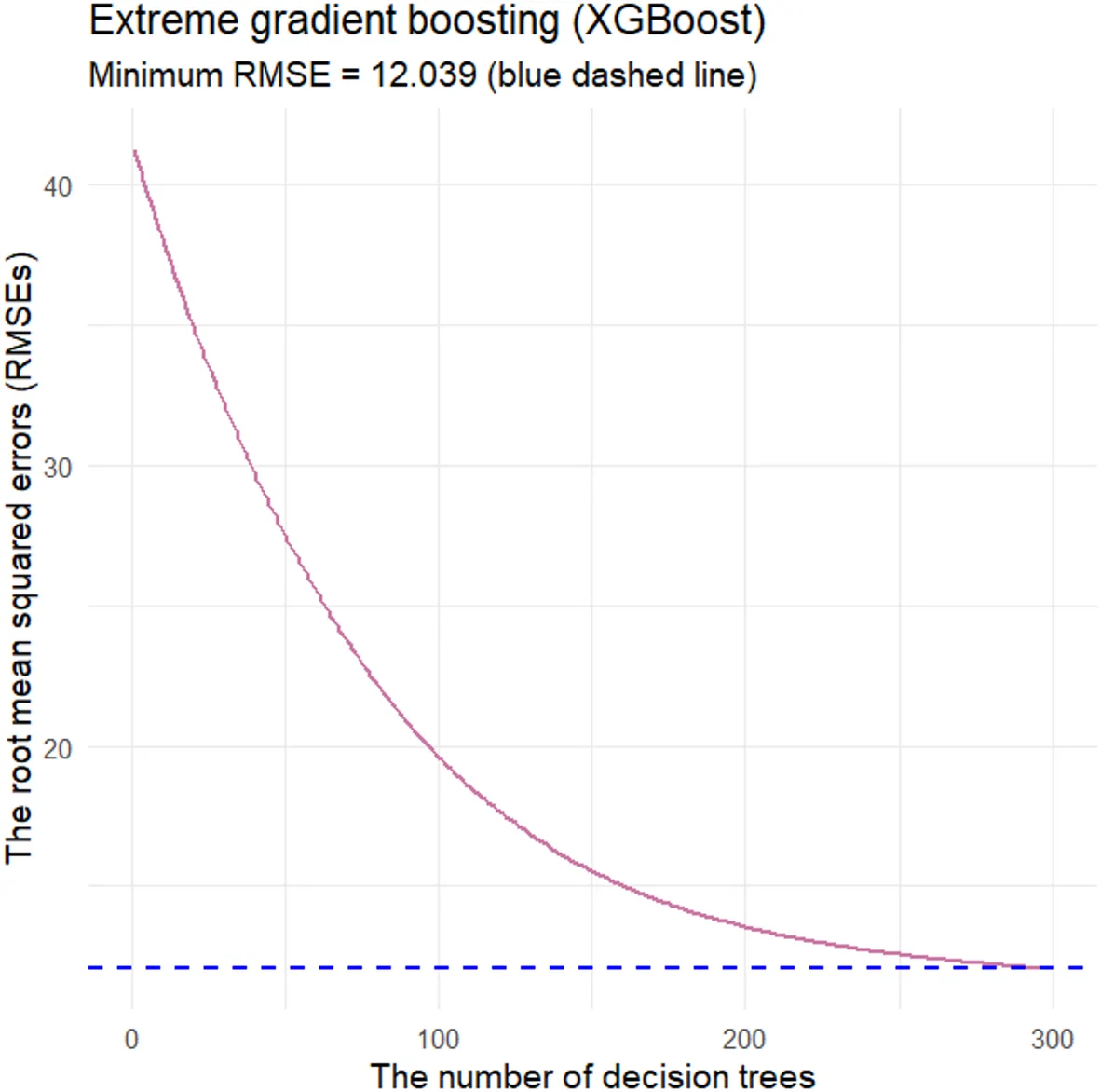
The values of root mean squared errors (RMSEs) in relation to the number of trees in XGBoost.

Figure 10 illustrates a similar plot containing the values of the root mean squared errors (RMSEs) for each decision tree in the context of XGBoost (with nrounds = 300). This figure demonstrates that the RMSEs stabilize across the updated decision trees, indicating that a total of 300 decision trees is adequate. The minimum value of RMSEs for XGBoost is obtained as 12.039 (refer to the blue dashed line in Fig. 10). Table 7 shows the values of the importance measures for the attributes in the training set using RFR, XGBoost, and SVR, respectively. The technique we employ in RFR randomly shuffles the values of each attribute and utilize the percentage increase in the out-of-bag MSE as a measure of the importance of attributes in the training set. In a similar manner, the gain in XGBoost indicates the extent to which the attributes reduce the value of the loss function, which is the MSE in our context. Permutation feature importance is utilized in SVR to assess the contribution of each attribute to the predictive performance of the model in the training set. Table 7 shows that the metrics employed in RFR, XGBoost, and SVR exhibit consistent results, indicating that DR, DNI, and TR are the most important attributes in predicting the positive values of AP. These features play a significant role not only in modeling the positive values of AP but also in modeling the structural zeros that are associated with AP. In this regard, the importance of these attributes for PV power forecasting was elaborated in the subsection “Data Preprocessing”.

It is important to note that the similarity matrix employed in SVR and the decision trees utilized in RFR and XGBoost possess the ability to capture the interactions among the attributes. Consequently, no interaction terms are incorporated in the non-parametric techniques. However, the parametric models GR, LNR, and WR lack this capability. Therefore, in addition to the attributes in Eq. 6, we use a set of interaction terms in our parametric models. By undertaking this approach, our objective is to enhance the performances of our parametric models within both the training and testing sets.

**Table 7:** The values of importance measures for the attributes in the context of RFR, XGBoost, and SVR.

Attributes	RFR: Percentage increase in MSE	XGBoost: Gain	SVR: Permutation feature importance
T	75.869	0.015	1.090
RH	37.101	0.020	1.065
WS	21.153	0.016	1.082
DR	**429.523**	**0.547**	**1.444**
DNI	**168.336**	**0.124**	**1.133**
TR	**334.781**	**0.210**	**1.839**
WD	16.573	0.012	1.040
DP	27.877	0.006	1.071
CC	37.929	0.013	1.059
t	18.742	0.008	1.029
sin($\:\frac{2\pi\:t}{P}$)	24.151	0.007	1.040
cos($\:\frac{2\pi\:t}{P}$)	55.456	0.020	1.114

Significant values are in [bold].

In this regard, six interaction terms have been incorporated into each parametric model, which are T $\:\times\:$ DNI, RH $\:\times\:$ DNI, T $\:\times\:$ DR, RH $\:\times\:$ DR, T $\:\times\:$ WS, and RH $\:\times\:$ WS. Table 8 displays the maximum likelihood estimates (with the corresponding values of the test statistics between brackets), the (minus) log likelihood (-LL), the Akaike information criterion (AIC) and Bayesian information criterion (BIC) values obtained by GR, LNR, and WR, respectively. Each of the parametric models in Table 8 has a training time of less than one second. According to the findings shown in Table 8, the parametric model that most accurately represents the training set for positive values is identified as WR. Because this model produces smaller minus log likelihood, AIC, and BIC values compared to the other two models. Nevertheless, this conclusion is derived based on classical statistical inference on only positive values. Performance comparisons of both the parametric and non-parametric hurdle models for predicting the values of the target variable AP are carried out using a series of metrics on the testing set, which will be discussed in the next subsection.

**Table 8:** The maximum likelihood estimates (with the corresponding values of the test statistics between brackets), the (minus) log likelihood, and AIC and BIC values obtained by GR, LNR, and WR.

Effects	Gamma regression		Log-normal regression		Weibull Regression	
	β (t-value)	SE	β (z-value)	SE	β (z-value)	SE
(Intercept)	0.911** (2.606)	0.350	-0.727 (-1.432)	0.508	1.434*** (4.146)	0.346
T	-0.001 (-0.018)	0.017	0.020 (0.815)	0.025	-0.012 (-0.688)	0.017
RH	0.001 (0.105)	0.004	0.009 (1.784)	0.005	-0.003 (-0.766)	0.004
WS	0.093*** (10.568)	0.009	0.133*** (10.472)	0.013	0.080*** (9.581)	0.008
DR	-0.001** (-2.919)	0.001	-0.002** (-2.824)	0.001	-0.001 (-2.313)	0.001
DNI	0.001 (1.798)	0.001	0.001 (1.254)	0.001	0.001 (1.635)	0.001
TR	0.003*** (91.233)	0.001	0.003*** (73.909)	0.001	0.003*** (77.560)	0.001
WD	-0.001*** (-5.331)	0.001	-0.001*** (-4.584)	0.001	-0.001*** (-4.405)	0.001
DP	-0.005 (-0.259)	0.018	-0.013 (-0.529)	0.025	0.007 (0.382)	0.017
CC	0.001 (0.785)	0.001	-0.001 (-0.798)	0.001	0.001 (1.516)	0.001
t	-0.001*** (-3.809)	0.001	0.001 (0.419)	0.001	-0.001*** (-4.599)	0.001
sin($\:\frac{2\pi\:t}{P}$)	-0.057*** (-5.682)	0.010	-0.023 (-1.567)	0.014	-0.055*** (-5.604)	0.010
cos($\:\frac{2\pi\:t}{P}$)	-0.162*** (-9.930)	0.016	-0.079*** (-3.353)	0.024	-0.167*** (-10.496)	0.016
T $\:\times\:$ DNI	-0.001** (-2.770)	0.001	0.001** (3.068)	0.001	-0.001*** (-3.588)	0.001
RH $\:\times\:$ DNI	0.001*** (15.524)	0.001	0.001*** (8.829)	0.001	0.001*** (11.701)	0.001
T $\:\times\:$ DR	0.001*** (3.729)	0.001	0.001 (0.123)	0.001	0.001*** (3.867)	0.001
RH $\:\times\:$ DR	-0.001*** (-6.587)	0.001	-0.001*** (-4.519)	0.001	-0.001*** (-6.569)	0.001
T $\:\times\:$ WS	-0.001*** (-7.298)	0.001	-0.002*** (-6.541)	0.001	-0.001*** (-7.077)	0.001
RH $\:\times\:$ WS	-0.001*** (-14.229)	0.001	-0.002*** (-13.316)	0.001	-0.001*** (-13.218)	0.001
Statistics						
-LL	52,113.0		54,396.3		51,662.7	
AIC	104,265.9		108,832.7		103,365.4	
BIC	104,415.9		108,982.6		103,515.4	

Note: * : *p*-value < 0.05, **: *p*-value < 0.01, and ***: *p*-value < 0.001.

### Fitted values and performance evaluation in the testing set

We obtain the fitted values, and consequently, evaluate the predictive performance of each hurdle model under consideration using the testing set (*n* = 6,589). As mentioned, the zero component of the hurdle models was modeled using BLR, RFC, and SVC. The positive component was examined using the parametric models GR, LNR, and WR and the non-parametric ML techniques RFR, XGBoost, and SVR. Notably, this yields 3 $\:\times\:$ 6 = 18 distinct hurdle models. In the following two subsections, we provide a detailed explanation of how to derive the fitted values for each of the 18 hurdle models and evaluate their predictive performance, respectively.

#### Obtaining fitted values for hurdle models

To derive the fitted values for the hurdle models, we initially acquire those for BLR, RFC, and SVC when modeling the structural zeros in the testing set, that is, $\:\left(1-{\widehat{\pi\:}}_{i}\right)$ for i = 1, 2, … 6,589. Then, we obtain the fitted values using RFR, XGBoost, SVR, GR, LNR, and WR when modeling positive values in the testing set, that is, $\:{\widehat{y}}_{i}$ = E($\:{y}_{i}$|$\:{y}_{i}$ > 0 ) for i = 1, 2, … 6,589. These two types of fitted values are integrated within the framework of hurdle models by multiplying the corresponding values, that is, $\:\left(1-{\widehat{\pi\:}}_{i}\right)$ $\:\times\:$ $\:{\widehat{y}}_{i}$ for i = 1, 2, … 6,589. We perform comparisons of the hurdle models regarding their ability to predict the values of the target variable AP by utilizing four different metrics, each of which employs the integrated fitted values above.

#### Predictive performance evaluation

The effectiveness of the BLR, RFC, and SVC techniques employed to model structural zeros was evaluated using the confusion matrices on the testing set. Table 9 displays the confusion matrices between the actual and predicted values based on BLR, RFC, and SVC for the testing set. This table indicates that each of the BLR, RFC, and SVC techniques demonstrates satisfactory performance in the testing set according to the CCR. Similar to the training set results in Table 5, RFC produces slightly higher accuracy (CCR = 0.992) compared to BLR (CCR = 0.981) and SVC (CCR = 0.970). The Kappa coefficient for RFC is determined to be K = 0.985, indicating a robust model for forecasting the structural zeros in the testing set.

**Table 9:** The confusion matrices between the actual and predicted values based on BLR, RFC, and SVC for the testing set.

	Predicted values	Status	
		0: Not a structural zero	1: Structural zero
BLR	0	3,398	14
	1	109	3,068
RFC	0	3,478	21
	1	29	3,061
SVC	0	3,319	9
	1	188	3,073

The list of the four metrics used to assess the predictive performance of the 18 hurdle models is as follows: mean squared error (MSE), mean absolute error (MAE), mean absolute scaled error (MASE), and the coefficient of determination ($\:{R}^{2}$). The evaluation metrics are defined as follows:

$$MSE = \:\frac{1}{\mathrm{n}}\sum\:_{\mathrm{i}=1}^{\mathrm{n}}{\left({\mathrm{y}}_{\mathrm{i}}\:-{\widehat{\mathrm{y}}}_{\mathrm{i}}\right)}^{2},$$

$$MAE =\:\frac{1}{\mathrm{n}}\sum\:_{\mathrm{i}=1}^{\mathrm{n}}\left|{\mathrm{y}}_{\mathrm{i}}\:-{\widehat{\mathrm{y}}}_{\mathrm{i}}\right|,$$

$$MASE = \:\frac{\mathrm{M}\mathrm{A}\mathrm{E}}{\frac{1}{\mathrm{n}-1}\sum\:_{\mathrm{i}=2}^{\mathrm{n}}\left|{\mathrm{y}}_{\mathrm{i}}\:-{\mathrm{y}}_{\mathrm{i}-1}\right|},$$

7 $$\:{R}^{2}=\:\frac{{\left[{\sum\:}_{i=1}^{n}({y}_{i}-\stackrel{-}{y})({\widehat{y}}_{i}-\stackrel{-}{\widehat{y}})\right]}^{2}}{\sqrt{{\sum\:}_{i=1}^{n}{({y}_{i}-\stackrel{-}{y})}^{2}{\sum\:}_{i=1}^{n}{({\widehat{y}}_{i}-\stackrel{-}{\widehat{y}})}^{2}}},$$

where $\:{\mathrm{y}}_{\mathrm{i}}$ and $\:{\widehat{\mathrm{y}}}_{\mathrm{i}}$ denote the observed and fitted values for the testing data with the corresponding means $\:\stackrel{-}{y}$ and $\:\stackrel{-}{\widehat{y}}$, respectively. Since the AP is measured in kW, MAE and RMSE are expressed in kW, whereas MASE and R² are unit-free due to scaling.

Table 10 displays the values of these metrics for a set of single-stage models (RFR, XGBoost, SVR, and LSTM) and each hurdle model under consideration. Note that the single-stage GR, LNR, and WR cannot be utilized in this context, as these models are designed to operate exclusively with positive values (and not with zeros). The findings concerning RFR versus its two-stage hybrid counterparts (BLR-RFR, RFC-RFR, and SVC-RFR) and XGboost versus its counterparts (BLR-XGBoost, RFC-XGBoost, and SVC-XGBoost) were notably similar, where as the two-stage BLR-SVR, RFC-SVR, and SVC-SVR exhibited enhanced performance over SVR across all predictive performance metrics. Since LSTM is sensitive to the scale of the data, it was employed on the (min-max) standardized data. It should be noted that standardization also alters the scale of the some of the performance metrics above. Therefore, the comparison of LSTM with the other techniques was conducted following a back-transformation procedure. A sigmoid function was employed within LSTM to restrict outputs to non-negative values in line with the characteristics of the data. Since the training data contain a sufficient number of observations, a look-back window of 100 timesteps was employed. Further details regarding the single-stage LSTM procedure will be provided upon request.

Based on the results presented in Table 10, the best predictive performances were achieved when the positive component was modeled using either RFR or XGBoost. In this sense, for example, BLR-RFR (MSE = 67.425 kW, MAE = 3.974 kW, MASE = 0.615, $\:{R}^{2}$ = 0.899) and BLR-XGBoost (MSE = 68.815 kW, MAE = 4.052 kW, MASE = 0.627, $\:{R}^{2}$ = 0.899) performed better than BLR-SVR (MSE = 96.191 kW, MAE = 5.864 kW, MASE = 0.907, $\:{R}^{2}$ = 0.872). It appears that the hurdle models with the ML techniques perform better than that with the parametric models. All the hurdle models exhibit satisfying performance in terms of explaning the variance of the target variable AP. It is important to note that even the least effective model RFC-LNR manages to account for more than 86% (with $\:{R}^{2}$ = 0.861) of the variance in AP. This percentage reaches almost 90% (with $\:{R}^{2}$ = 0.899) for BLR-RFR and RFC-RFR.

**Table 10:** The values of the predictive performance metrics for the 18 hurdle models under consideration.

	MSE	MAE	MASE	$\:{R}^{2}$
RFR	70.733	3.865	0.223	0.889
**XGBoost**	**69.595**	**4.091**	**0.633**	**0.898**
SVR	147.021	10.486	1.622	0.870
LSTM	73.959	4.442	0.685	0.893
**BLR-RFR**	**67.425**	**3.974**	**0.615**	**0.899**
**BLR-XGBoost**	**68.815**	**4.052**	**0.627**	**0.899**
BLR-SVR	96.191	5.864	0.907	0.872
BLR-GR	80.247	4.415	0.683	0.882
BLR-LNR	98.194	4.997	0.773	0.862
BLR-WR	82.526	4.444	0.687	0.881
**RFC-RFR**	**68.416**	**4.094**	**0.633**	**0.899**
**RFC-XGBoost**	**72.028**	**4.281**	**0.662**	**0.898**
RFC-SVR	102.899	6.443	0.996	0.872
RFC-GR	76.685	4.461	0.690	0.882
RFC-LNR	95.715	4.967	0.768	0.861
RFC-WR	79.565	4.470	0.691	0.881
**SVC-RFR**	**67.542**	**3.969**	**0.614**	**0.898**
**SVC-XGBoost**	**69.252**	**4.075**	**0.630**	**0.898**
SVC-SVR	96.511	5.805	0.898	0.871
SVC-GR	80.038	4.401	0.681	0.882
SVC-LNR	97.656	4.981	0.770	0.862
SVC-WR	82.357	4.428	0.685	0.881

Significant values are in [bold].

## Discussion, conclusion, and limitation

This study presents an analysis of PV power forecasting data with excessive structural zeros using a hybrid modeling strategy. The analysis divided the data into two parts: a training set (the first 80% of the data) and a testing set (the remaining 20% of the data). The study examined both the training and testing sets using two-stage hurdle models. In the first stage, BLR, RFC, and SVC were employed to model the structural zeros in the data. In the second stage, the remaining positive values were analyzed using the parametric models GR, LNR, and WR, as well as the non-parametric ML techniques RFR, XGBoost, and SVR.

The training and testing performances of BLR, RFC, and SVC were evaluated according to their ability to identify structural zeros. In the training set, although each of these models exhibited a high level of accuracy in terms of the correct classification rate (CCR), RFC (CCR = 0.992) performed slightly better than BLR and SVC (both with CCR = 0.980). The execution times for RFC (20.40 s) and SVC (35.21 s) were significantly shorter than that for BLR (41.30 min) (see Table 4). In the testing set, similar results were achieved with respect to the level of accuracy. Namely, RFC (CCR = 0.992) provided a slightly higher accuracy when compared to BLR (CCR = 0.981) and SVC (CCR = 0.970). The Kappa coefficients (K) calculated from the confusion matrices of the actual and predicted values in Table 5 (training set) and Table 9 (testing set) indicated that each technique is highly effective for predicting the structural zeros in the PV power forecasting data. In this sense, for example, RFC produced high Kappa coefficient values, specifically, K = 0.984 and K = 0.985 for the training and testing sets, respectively. When analyzing the positive values in the training set, the metrics employed in RFR, XGBoost, and SVR exhibited consistent results, indicating that DR, DNI, and TR are the most important attributes in predicting the values of AP (see Table 7). The WR model was chosen as the best model among the parametric models when modeling the positive values in the training set, as it produced the smallest -LL (51,662.7), AIC (103,365.4), and BIC (103,515.4) values compared to GR and LNR.

The hurdle modeling strategy achieved the highest predictive accuracy when the positive component was modeled using either RFR or XGBoost (see Table 10). In this regard, BLR-RFR (MSE = 67.425 kW, MAE = 3.974 kW, MASE = 0.615, $\:{R}^{2}$ = 0.899) and BLR-XGBoost (MSE = 68.815 kW, MAE = 4.052 kW, MASE = 0.627, $\:{R}^{2}$ = 0.899) demonstrated superior performance compared to BLR-SVR (MSE = 96.191 kW, MAE = 5.864 kW, MASE = 0.907, $\:{R}^{2}$ = 0.872). Additionally, the hurdle models using the ML techniques RFR, XGBoost, and SVR for modeling positive values in the data outperformed using the parametric alternatives GR, LNR, and WR. All hurdle models exhibited satisfactory performance in explaining the variance of the target variable AP. It is important to note that even the least effective RFC-LNR model managed to account 86% of the variance in AP. This rate reached to almost 90% when the positive component was modeled using RFR or XGBoost regardless of the technique used for modeling the structural zeros in the data. This rate almost reached 90% when the positive component was modeled using either RFR or XGBoost, regardless of the technique employed to model the structural zeros in the data.

The effectiveness of RFR and XGBoost in the single-stage settings highlights their potential for two-stage hurdle modeling. This is primarily attributed to their capacities to capture intricate non-linear relationships among the variables in the presence of outliers when compared to their parametric alternatives. Note that, the structural zeros are *not* modeled separately from the positive values in the single-stage settings. Therefore, these structural zeros render many positive values of the target variable AP as outliers when using the single-stage techniques. In the literature, both RFR and XGBoost are considered as robust techniques when dealing with non-linearity^67–70^ and outliers^71–73^ in the data. Conversely, the parametric models GR, LNR, and WR are sensitive to outliers due to their functional characteristics^74–77^. Additionally, these models are not defined for non-positive values, and do not capture the non-linear relationships between the variables in the data. Consequently, hurdle models incorporating RFR and XGBoost can be asserted to produce superior modeling performance compared to those utilizing GR, LNR, and WR. It is important to note that while both RFR and XGBoost employ decision trees for modeling the target variable AP there exist notable differences in their implementations. While RFR models the data by capturing the underlying relationships between the variables through independent decision trees, XGBoost constructs decision trees in a sequential manner, treating them as interdependent models. Moreover, the predictive performance of SVR is directly related to the capacity of the kernel function employed for capturing the intricate relationships among the variables in the data. The hurdle models containing SVR for modeling positive component with a Gaussian kernel exhibited inferior predictive performance relative to RFR and XGBoost (see Table 10).

Despite its promising results, the proposed framework has several limitations that should be acknowledged. Below, we address four main limitations that we deem significant. One of the key limitations of our investigation is that the analysis is based on the data from a single photovoltaic plant, which may limit the generalizability of the findings to other geographical regions, climatic conditions, or system configurations. Another important limitation is that hybrid models, which are part of the two-stage model family, usually demand more observations than the traditional parametric models or ML techniques. Therefore, employing these models with small samples may lead to overfitting problem. Third limitation is that hybrid models exhibit greater memory consumption and longer training time when compared to the classical single-stage models. The convergence of these models requires more time compared to classical techniques, particularly when dealing with large datasets. Last but not least, the three classification techniques implemented in this study (BLR, RFC, and SVC) produced comparable and satisfactory predictive performances when modeling the structural zeros in the data. As mentioned, we set the tuned hyperparameters for RFC and SVC identically to those of RFR and SVR, respectively (see Table 6). In this regard, the use of different hyperparameters may yield different results. Additionally, since BLR produced results comparable to those of RFC and SVC, and furthermore, its execution time was considerably longer than that of the other techniques, the robustness of the findings in relation to the choice of the cut-off value or prior selection was not investigated. In this regard, undertaking sensitivity analysis for both BLR and the ML techniques RFC, SVC, RFR, and XGBoost for PV power forecasting might be seen as a potential subject for future research.

## Supplementary Information

Below is the link to the electronic supplementary material.

Supplementary Material 1

Supplementary Material 2

## Data Availability

The data used in this study are available from the corresponding author upon reasonable request.
